# Exploration of a Large Virtual Chemical Space: Identification of Potent Inhibitors of Lactate Dehydrogenase-A against Pancreatic Cancer

**DOI:** 10.1021/acs.jcim.2c01544

**Published:** 2023-01-16

**Authors:** Horrick Sharma, Pragya Sharma, Uzziah Urquiza, Lerin R. Chastain, Michael A. Ihnat

**Affiliations:** † Department of Pharmaceutical Sciences, College of Pharmacy, Southwestern Oklahoma State University, Weatherford, Oklahoma73096, United States; ‡ Department of Biological Sciences, Southwestern Oklahoma State University, Weatherford, Oklahoma73096, United States; § Department of Pharmaceutical Sciences, College of Pharmacy, University of Oklahoma Health Sciences Center, Oklahoma City, Oklahoma73117, United States

## Abstract

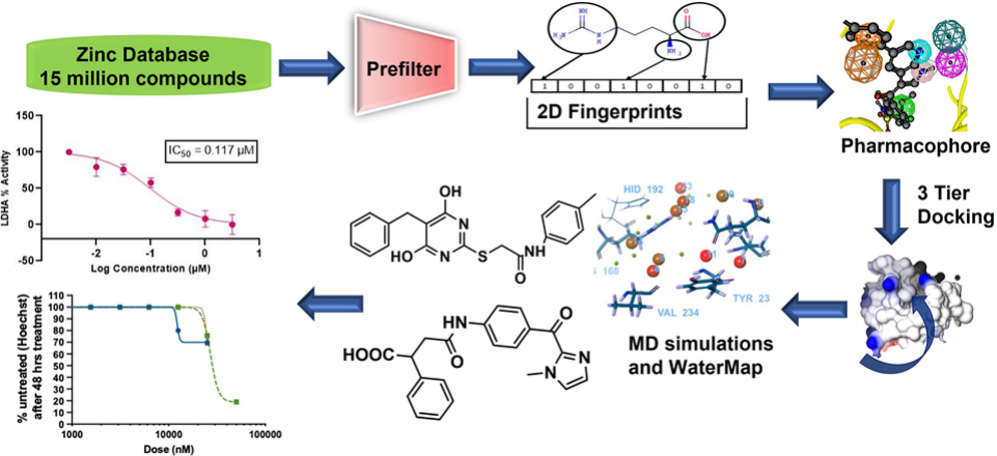

It is imperative to explore the gigantic available chemical space to identify new scaffolds for drug lead discovery. Identifying potent hits from virtual screening of large chemical databases is challenging and computationally demanding. Rather than the traditional two-dimensional (2D)/three-dimensional (3D) approaches on smaller chemical libraries of a few hundred thousand compounds, we screened a ZINC library of 15 million compounds using multiple computational methods. Here, we present the successful application of a virtual screening methodology that identifies several chemotypes as starting hits against lactate dehydrogenase-A (LDHA). From 29 compounds identified from virtual screening, 17 (58%) showed IC_50_ values < 63 μM, two showed single-digit micromolar inhibition, and the most potent hit compound had IC_50_ down to 117 nM. We enriched the database and employed an ensemble approach by combining 2D fingerprint similarity searches, pharmacophore modeling, molecular docking, and molecular dynamics. WaterMap calculations were carried out to explore the thermodynamics of surface water molecules and gain insights into the LDHA binding pocket. The present work has led to the discovery of two new chemical classes, including compounds with a succinic acid monoamide moiety or a hydroxy pyrimidinone ring system. Selected hits block lactate production in cells and inhibit pancreatic cancer cell lines with cytotoxicity IC_50_ down to 12.26 μM against MIAPaCa-2 cells and 14.64 μM against PANC-1, which, under normoxic conditions, is already comparable or more potent than most currently available known LDHA inhibitors.

## Introduction

**Figure 1:**
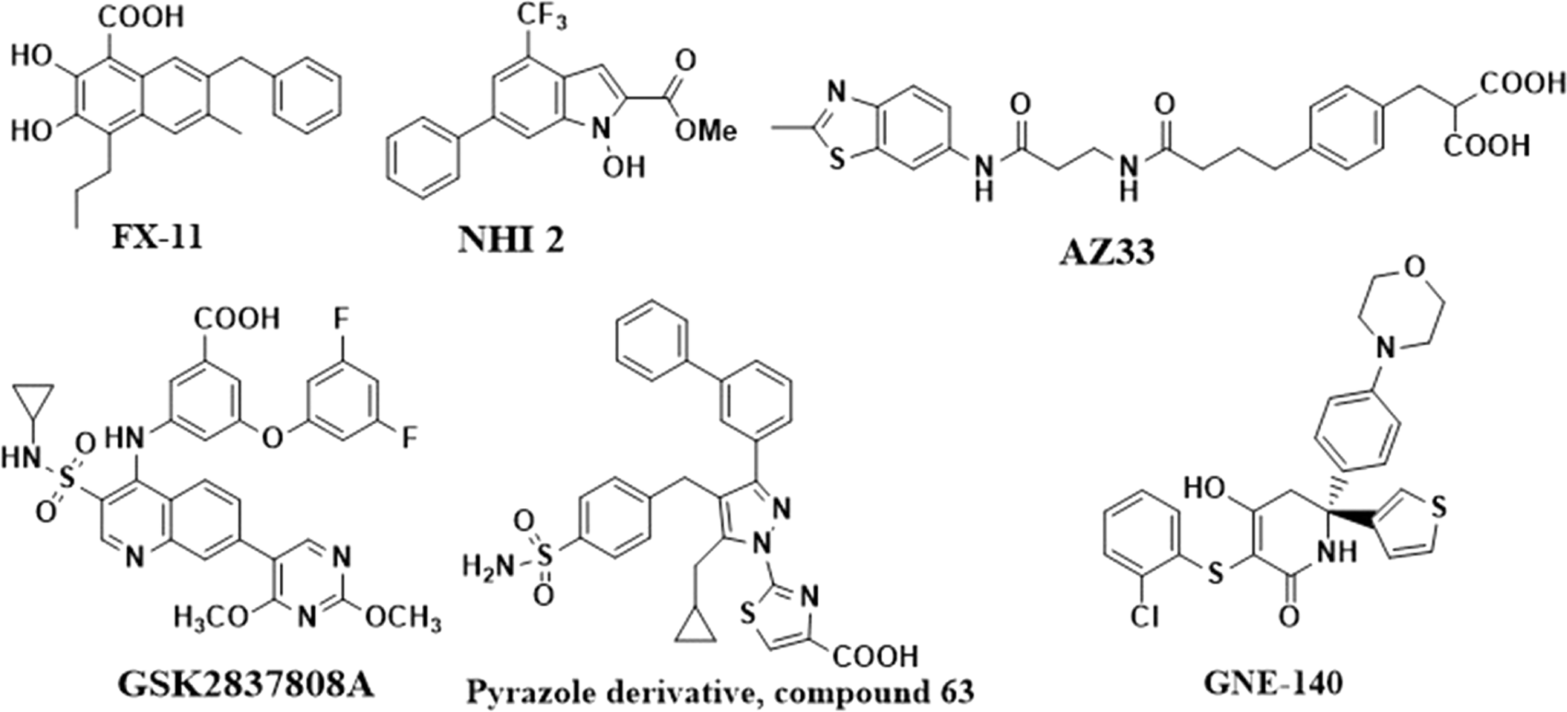
LDHA inhibitors reported in the literature.

Metabolic reprogramming has emerged as a hallmark of cancer cells.^1^ Most tumors, including pancreatic cancer, undergo a switch from oxidative phosphorylation (OXPHOS) to aerobic glycolysis (Warburg effect) and convert glucose to lactic acid for their survival and growth.^2,3^ In pancreatic ductal adenocarcinoma (PDAC) patients, glycolytic tumors represent a clinical subgroup with the shortest median survival in resectable and metastatic states.^4^ Glucose is an essential nutrient required for the growth of most tumors, including PDAC. KRAS mutations upregulate glucose transporter-1 and glycolytic enzymes, including lactate dehydrogenase-A (LDHA).^5^ LDHA executes the last step of aerobic glycolysis by catalyzing the conversion of pyruvate to lactic acid, an oncometabolite and metabolic fuel involved in the carcinogenesis and acidification of the tumor microenvironment.^6,7^**FX-11** is an LDHA inhibitor with a Ki of 8.0 μM, which, as a single agent, is shown to inhibit human pancreatic cancer xenografts.^8^ In addition to **FX-11**, a few LDHA inhibitors are reported, but only some (Figure 1) have demonstrated cellular activity.^9−14^ Further, the development of these molecules is hampered as the structures lack drug-like properties and have poor pharmacokinetic profiles. Thus, greater exploration of significantly sizeable chemical space is needed to discover new molecules targeting the Warburg effect for cancer therapy. Virtual screening can be used to find new chemotypes and may employ several methodologies, ranging from two-dimensional (2D) and three-dimensional (3D) ligand-based approaches to structure-based docking and molecular dynamics (MD) simulations.^15,16^ Ideally, virtual screening should rank-order compounds based on their binding affinity, and enrichment metrics should be maximized to generate an enriched subset of compounds at each stage, progressing to experimental validation.^17^

**Figure 2:**
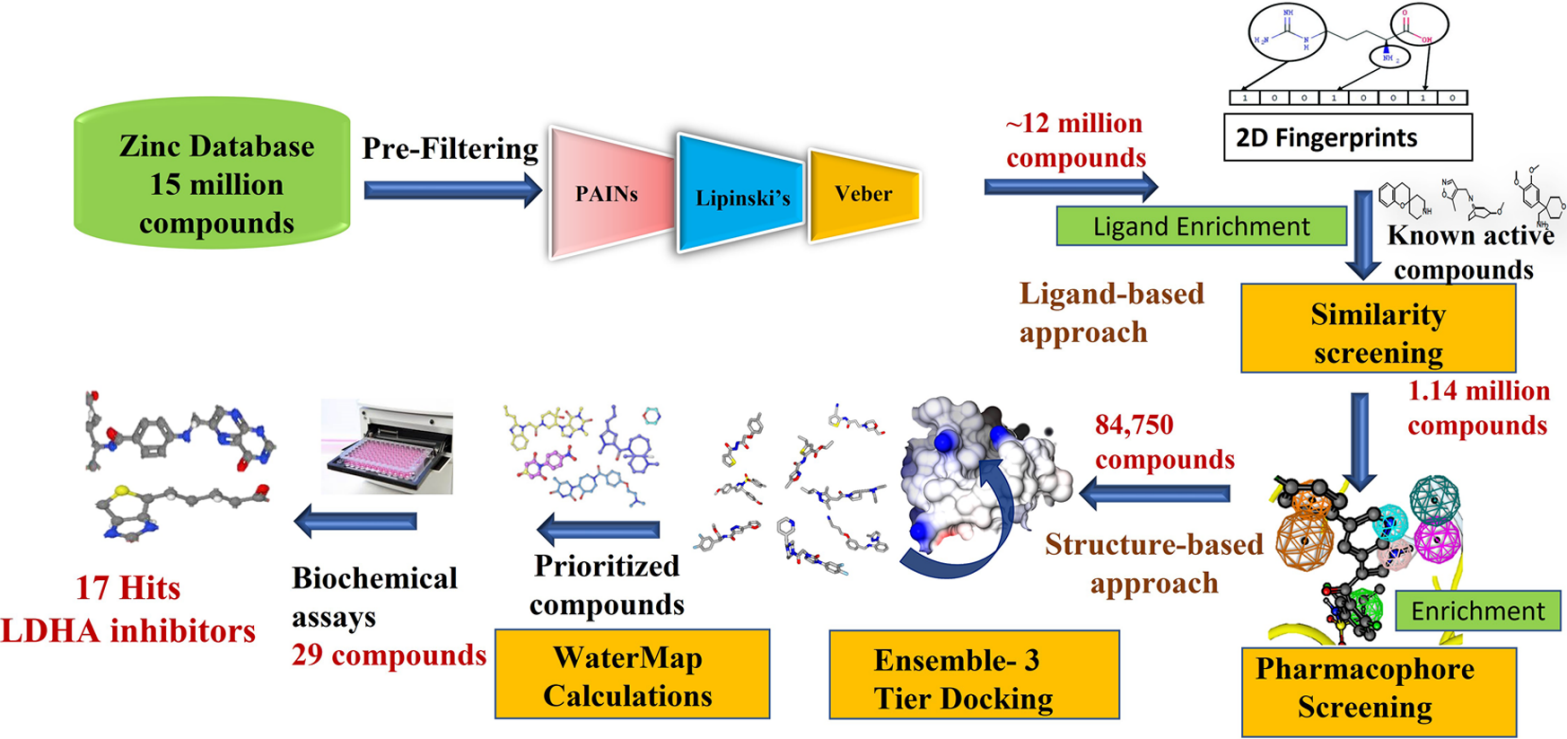
Virtual screening workflow identifying several hits as novel LDHA inhibitors. A library of 15 million compounds was screened to remove the reactive functional group, and compounds conforming to Veber and Lipinski’s rule were selected. The filtered compounds were then screened through 2D Fingerprint searches, followed by pharmacophore and molecular docking. WaterMap calculations were used for posing selection, and 29 compounds were tested in biochemical assays. The screening identified 17 hits, the binding poses of which were confirmed with MD simulations.

In the present work, we report virtual screening (Figure 2), leading to the discovery of compounds that inhibit LDHA with low micromolar and sub-micromolar concentrations. We screened a ZINC library of ∼15 million compounds using a combination of 2D fingerprint similarity searches, pharmacophore screening, and ensemble docking and carried out WaterMap calculations to determine critical residues that could be targeted for LDHA inhibition. From 29 compounds selected for testing, 17 compounds showed LDHA inhibitory activity < 63 μM. Interestingly, the most potent hit, **ZINC13469319**, inhibits LDHA with an IC_50_ of 117 nM. Selected compounds also reduced intracellular lactate production in MIAPaCa-2 cells and showed cytotoxicity against human PANC-1 and MIAPaCa-2 and mouse FC1199 pancreatic cancer cell lines with cell viability IC_50_ down to 12.26 μM against MIAPaCa-2 and 14.64 μM against PANC-1. We also explored the binding poses of these novel LDHA inhibitors with 50 ns MD simulations and molecular mechanics/generalized born surface area (MM-GBSA) binding free-energy calculations.

## Results and Discussion

### Similarity-Based 2D Fingerprint Screening

#### Creation of Database

Approximately 15 million compounds (14,955,127 compounds) from the ZINC database were passed through REOS and PAINS filters (available in Canvas, Schrödinger) to assess clean drug-like compounds.^18,19^ The resulting compounds were further filtered using Lipinski’s and Veber’s rules. Specifically, to access compounds with lead-like properties, we kept the molecular weight within 150–450 g/mol, and log *P* was set to ≤5.0. This prefiltering resulted in 12,689,737 compounds for screening.

#### Preparation of Active and Decoy Subset

Enrichment is a measure of identifying known active compounds from decoy molecules. To evaluate retrospective performance in virtual screening, we performed ligand enrichment using target-specific decoys and a subset of known active ligands. We retrieved 123 LDHA inhibitors with an IC_50_ or Ki threshold of <20 μM from the ChEMBL database.^20^ Hierarchical clustering of 123 actives using the average-linkage as the distance metrics with MOLPRINT2D fingerprint and Tanimoto index resulted in 17 clusters.^21^ For selection, we set a threshold of a maximum of three compounds per cluster, and then, based on the diversity and potency, 28 inhibitors, shown in the Supporting Information, (Table S1) were chosen as actives for validation studies. For chiral compounds, the more active stereoisomer was considered for the study. The 28 actives were used to create target-specific decoys using the ZINC DUD-E and Schrödinger’s decoy dataset.^22^ Decoys were selected to mimic ligands physically in terms of the molecular weight, *c* log *P*, number of H-bond donors and acceptors, and the presence of rotatable bonds. However, since the decoys are not active, compounds that are topologically dissimilar to the active ligands were selected. DecoyFinder, a python GUI application, was used to generate decoys.^23^ The minimum and maximum decoys/ligands ratios were set to 4 and 36, respectively. In addition, we obtained functional decoys from the ChEMBL database. A decoy set of 1170 compounds was created and used for the study.

#### 2D Molecular Fingerprints

2D molecular fingerprints, including structural keys and hashed fingerprints, were generated with the Canvas package in the Schrödinger suite. Molecular fingerprints represent structural features and properties in binary vectors for computational programs.^24^ We generated dendritic, MOLPRINT2D, and radial hashed fingerprints and MACCS structural keys from the structures of the 28 LDHA inhibitors and carried out an intra-dataset validation using 1198 compounds, including 28 actives and 1170 decoys. Enrichment was computed based on the similarity between the fingerprints of a dataset molecule and the active query molecule. The dataset was then ranked based on the decreasing order of the similarity scores. MACCS fingerprint scans for the presence (or absence) of specific structural fragments from a predefined list (containing a subset of 166 keys) and accordingly assigns ones or zeros as binary vector elements.^25^ Hashed fingerprints do not use a predefined key but split the entire molecule into fragments and employ a “hash” function to convert the fragments into numerical values. Fingerprints were encoded by hashing each chemical fragment into the 232 size space and storing only the “on” bits to reduce the collision rate.^26^ Hashed fingerprints are differentiated based on the paths the fragments are connected within the molecule.^24,27^ In the dendritic fingerprint, the structure is decomposed into fragments grown in linear and branched directions with up to five bonds per path. In contrast, in radial fingerprints, the fragments are expanded radially from each heavy atom over a series of iterations. MOLPRINT2D is similar to the radial fingerprint, but each heavy atom in the molecule is encoded in an environment comprising all other MOL2 heavy atoms within a 2-bond distance. The SYBYL atom types are encoded as a string and converted to an integer, representing a bit in the fingerprint. We used the daylight atom-typing scheme for all hashed fingerprints except MOLPRINT2D. The daylight scheme represents the molecule as a graph, with nodes representing atoms and edges representing bonds. The atoms are differentiated based on the atom number, valence, formal charge, and the number of hydrogen- and nonhydrogen connections.^28^ For MOLPRINT2D, the SYBYL Mol2 atom type was used. We used the “bit” scaling and the “Tanimoto” index measure to calculate the similarity (SIM) scores, and the dataset was ranked in decreasing order of the similarity scores.^29,30^

#### Data Fusion

We used similarity and group fusion methods and combined information from multiple sources to achieve greater predictiveness than that obtained from a single source of information.^31,32^ In the similarity fusion, we combined SIM scores obtained from one reference query structure with “*n*” different similarity measures. In the group fusion method, we combined the SIM scores from “*y*” reference structures and a single similarity measure.

#### Fusion Rules

For enrichment of the database, the similarity and group fusion methods were combined using “MAX” and “SUM” fusion rules.^32^ The MAX rule considers and ranks the database compounds based on the highest SIM scores. The Max rule is represented by the equation below. The SIM scores were obtained from 1 – *y*th reference structure using 1 – *n* similarity measures. The measure that gave the maximum similarity with the active query compound was identified.
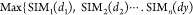


In the “SUM” fusion rule, the similarity scores from the “1 – *n*” measures and “1 – *y*” reference structure were combined to give the “SUM” score, which was used to rank order the database. The SUM rule is represented by
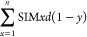


#### Enrichment Methods

We assessed enrichment with three different virtual screening strategies. First, we used each of the 28 actives individually in the query with four fingerprints: radial, dendritic, MOLPRINT2D, and MACCS. Thus, we evaluated (method A) 112 screening combinations to rank the validation dataset based on their Tanimoto similarity scores. In another strategy, method B, we applied MAX and SUM rules and fused the radial, dendritic, MOLPRINT2D, and MACCS similarity scores from each of the 28 actives. Fifty-six fusion scores were generated to assess enrichment. In another approach, method C, we took all 28 actives together as a query for screening. Thus, for each fingerprint, 28 similarity scores were computed, which were then processed using MAX and SUM fusion rules for ranking. Finally, eight fusion scores were used for validation. Since these studies resulted in an enormous volume of data, only the final best models will be represented.

We evaluated three screening methods and calculated the enrichment factor (EF), assaying the top 1, 5, and 10% compounds. EF is the concentration of the annotated ligands among the top-scoring hits compared to their concentration throughout the entire database.^33^ In other words, EF (*x*%) is the ratio of the probability of finding a hit in the top *x*% of the database to the hit rate obtained upon random searching of the entire database
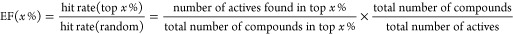
Among 112 similarity measures from method A, the best enrichment (shown in Table S2) was obtained using the MOLPRINT2D fingerprint and **CHEMBL1232973** as a reference compound. The similarity search identified six actives in the top 1%, 10 actives in the top 5%, and all 28 actives in the top 10% of the validation dataset. In method B, we used each of the 28 actives separately in the query for similarity search and fused the scores from the four fingerprints by applying the SUM and MAX rules. The ranking of MAX-fusion scores is shown in Table S3, while the SUM fusion scores ranking is shown in Table S4. Next, from the eight similarity searchers using method C, we observed maximum enrichment with MOLPRINT2D and MAX rule for data fusion (Table 1). The top-ranked models from methods A, B, and C are summarized in Table 2. Overall, the best enrichment for the top 1, 5, and 10% was achieved with the MOLPRINT2D fingerprint and “MAX” fusion rule (method C) with EF values of 42.8, 15, and 7.8, respectively.

**Table 1:** MAX-Fusion Scores-Based Ranking of the Top 10% of the Validation Dataset with MOLPRINT2D Fingerprint Using Method C

compound	active/decoy	activity (IC_50_/Ki) (μM)	MAX-fusion score	compound	active/decoy	activity (IC_50_/Ki) (μM)	MAX-fusion score
**CHEMBL3318527**	active	0.270	1	**CHEMBL3318444**	decoy		0.5
**CHEMBL3318535**	active	0.450	1	**CHEMBL3318447**	decoy		0.5
**CHEMBL3318538**	active	0.18	1	**CHEMBL3318452**	decoy		0.5
**CHEMBL3335792**	active	0.005	1	**CHEMBL3318453**	decoy		0.5
**CHEMBL2382401**	active	0.48	0.83871	**CHEMBL3318454**	decoy		0.5
**CHEMBL2382403**	active	0.71	0.83871	**CHEMBL3318462**	decoy		0.5
**CHEMBL2382404**	active	0.65	0.833333	**CHEMBL3318482**	decoy		0.5
**CHEMBL1688788**	active	15.70	0.8	**CHEMBL3318504**	decoy		0.5
**CHEMBL1688789**	active	19.80	0.8	**CHEMBL3318509**	decoy		0.5
**CHEMBL3359438**	active	0.36	0.791667	**CHEMBL3318515**	decoy		0.5
**CHEMBL3359439**	active	0.35	0.791667	**CHEMBL3318516**	decoy		0.5
**CHEMBL3359440**	active	0.030	0.791667	**CHEMBL2382390**	decoy		0.485714
**CHEMBL3581199**	active	0.030	0.766667	**CHEMBL3318483**	decoy		0.485714
**CHEMBL3581201**	active	0.015	0.724138	**CHEMBL3318480**	decoy		0.484848
**CHEMBL2430712**	decoy		0.76	**CHEMBL2059004**	decoy		0.483871
**CHEMBL3581200**	active	0.025	0.724138	**CHEMBL2430738**	decoy		0.483871
**CHEMBL2430727**	active	0.500	0.678571	**CHEMBL3318476**	decoy		0.483871
**CHEMBL2430733**	active	2.0	0.678571	**CHEMBL3318465**	decoy		0.482759
**CHEMBL2430734**	active	2.0	0.678571	**CHEMBL3358879**	decoy		0.48
**CHEMBL1688790**	active	4.70	0.666667	**CHEMBL3221028**	active	20.0	0.478261
**CHEMBL2430742**	decoy		0.653846	**CHEMBL3318422**	decoy		0.478261
**CHEMBL2382338**	decoy		0.636364	**CHEMBL3318428**	decoy		0.478261
**CHEMBL2430723**	decoy		0.62963	**CHEMBL3318429**	decoy		0.478261
**CHEMBL2382406**	decoy		0.612903	**CHEMBL3318430**	decoy		0.478261
**CHEMBL3318448**	decoy		0.608696	**CHEMBL3318432**	decoy		0.478261
**CHEMBL2382339**	decoy		0.6	**CHEMBL3318445**	decoy		0.478261
**CHEMBL3318481**	decoy		0.6	**CHEMBL3318450**	decoy		0.478261
**CHEMBL3335796**	active	5.10	0.590909	**CHEMBL3318456**	decoy		0.478261
**CHEMBL3318468**	decoy		0.586207	**CHEMBL3318459**	decoy		0.478261
**CHEMBL3318435**	decoy		0.583333	**CHEMBL3318461**	decoy		0.478261
**CHEMBL3318413**	decoy		0.578947	**CHEMBL3318467**	decoy		0.478261
**CHEMBL3764862**	active	19.50	0.576923	**CHEMBL3318502**	decoy		0.47619
**CHEMBL3318517**	decoy		0.571429	**CHEMBL3318506**	decoy		0.47619
**CHEMBL2430724**	decoy		0.566667	**CHEMBL2382391**	decoy		0.472222
**CHEMBL2430725**	decoy		0.566667	**CHEMBL2382392**	decoy		0.472222
**CHEMBL2382393**	decoy		0.5625	**CHEMBL2382395**	decoy		0.472222
**CHEMBL3318469**	decoy		0.555556	**CHEMBL3318479**	decoy		0.470588
**CHEMBL3359435**	decoy		0.555556	**CHEMBL2059006**	decoy		0.46875
**CHEMBL2430714**	decoy		0.551724	**CHEMBL2430740**	decoy		0.46875
**CHEMBL2430726**	decoy		0.551724	**CHEMBL2430718**	decoy		0.466667
**CHEMBL3318463**	decoy		0.55	**CHEMBL3318433**	decoy		0.466667
**CHEMBL3318478**	decoy		0.53125	**CHEMBL3318464**	decoy		0.466667
**CHEMBL3318417**	decoy		0.52381	**CHEMBL3358875**	decoy		0.464286
**CHEMBL3318441**	decoy		0.52381	**CHEMBL3317461**	decoy		0.458333
**CHEMBL3318458**	decoy		0.52381	**CHEMBL3318424**	decoy		0.458333
**CHEMBL2430722**	decoy		0.517241	**CHEMBL3318426**	decoy		0.458333
**CHEMBL2430739**	decoy		0.517241	**CHEMBL3318427**	decoy		0.458333
**CHEMBL2058999**	decoy		0.5	**CHEMBL3318446**	decoy		0.458333
**CHEMBL2059010**	decoy		0.5	**CHEMBL3318451**	decoy		0.458333
**CHEMBL3318414**	decoy		0.5	**CHEMBL3318455**	decoy		0.458333
**CHEMBL3318416**	decoy		0.5	**CHEMBL3318466**	decoy		0.458333
**CHEMBL3318418**	decoy		0.5	**CHEMBL3318503**	decoy		0.454545
**CHEMBL3318419**	decoy		0.5	**CHEMBL3318510**	decoy		0.454545
**CHEMBL3318421**	decoy		0.5	**CHEMBL3318512**	decoy		0.454545
**CHEMBL3318431**	decoy		0.5	**CHEMBL3318470**	decoy		0.451613
**CHEMBL3318438**	decoy		0.5	**CHEMBL3318471**	decoy		0.451613
**CHEMBL3318439**	decoy		0.5	**CHEMBL2430720**	decoy		0.448276
**CHEMBL3318440**	decoy		0.5	**CHEMBL3358880**	decoy		0.444444
**CHEMBL3318442**	decoy		0.5	**CHEMBL3358882**	decoy		0.444444
**CHEMBL3318443**	decoy		0.5	**CHEMBL3318425**	decoy		0.44

**Table 2:** Comparison of the Best Models from the Three Approaches Used in 2D Fingerprint Screening

		number of actives retrieved			enrichment factor (%)		
enrichment method	query compound	top 1%	top 5%	top 10%	top 1%	top 5%	top 10%
method A using MOLPRINT2D	**CHEMBL1232973**	6	10	28	21.4	7.1	10
method B (sum fusion)	**CHEMBL1232973** and **CHEMBL3764862**	5	8	13	14.2	8.5	4.6
method C (MOLPRINT2D and “MAX” fusion rule)	all 28 actives together used as query	12	21	22	42.8	15	7.8

To validate the best model obtained, we calculated performance metrics, including the Boltzmann-enhanced discrimination of the receiver operating characteristic (BEDROC) score and the area under the curve (AUC) for the receiver operation characteristics (ROC).^34^ ROC curve is an objective test that can distinguish if a given model is better than the others in selecting known actives and discarding the inactives. ROC is a plot of sensitivity (true-positive rate) and 1-specificity (false-positive rate).^35^ Sensitivity refers to the fraction of true-positive (actives) to all actives in the validation database, identified through the virtual screening methodology, and is represented by the following equation
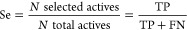
where TP is a true-positive, and FN is a false-negative. The sensitivity value can range between 0 (the model does not identify any actives) and 1 (when a model can identify all actives). Specificity is the fraction of true-negatives (inactive) being identified, and thus, discarded by the model. Specificity is represented by
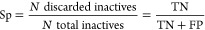
where TN is a true-negative, and FP is a false-positive. Specificity can also range from 0 (the model does not identify any inactive) to 1 (when a model can identify all inactives). The ROC plot resulting from the best enrichment from method C for the top 1% is shown in Figure S1. A higher AUC suggests that the model can discern actives from inactives, with AUC = 1 meaning that the model can correctly identify all actives and inactives (Se = Sp = 1). The plot shows an AUC (1%) of 0.95, which suggests 95% likely that a randomly selected active have a higher score than a randomly selected inactive. BEDROC is a weighted version of the AUC value, which focuses on the early enrichment of actives in the ROC curve and ranges from 0 to 1. The model showed a BEDROC (α = 20) score of 0.96. In BEDROC calculations, the use of α = 20 enables 80% of the BEDROC score to be computed from the top 8% of the ranked database.^34^ Therefore, we used this model to screen the 12,689,737 compounds but selected the top ∼10% ranked molecules, 1.14 million compounds, for pharmacophore modeling.

### Pharmacophore Screening

Compounds retrieved from the 2D fingerprint search were screened using pharmacophore models of LDHA. We built four pharmacophore models using ligand- and receptor-based approaches.^36,37^ We validated pharmacophore models by calculating the hit rates, sensitivity (true-positive rate), specificity (true-negative rate), ROC (receiver operating characteristic curve), and area under the ROC curve (AUC). To determine the effect of the number and type of active molecules in the test set and avoid bias, we included additional diverse LDHA inhibitors from the ChEMBL database to cover the “chemical space” more appropriately. We evaluated pharmacophore models with three test sets of active compounds. First, a pharmacophore model was developed using a set of 123 actives that were initially retrieved from the ChEMBL database. We then removed bulky and structurally very different molecules and used the remaining 104 actives to build the pharmacophore model. In another approach, we randomly selected 82 out of 123 active molecules for pharmacophore model generation. The decoys were kept to 1161 for each model. The X-ray structures were downloaded from the PDB weblink (www.rcsb.org). Two ligand-based pharmacophore models were developed (i) using bioactive ligand conformation of LDHA inhibitors from PDB ID codes: 4R69, 4ZVV, 4RLS, 5IXS, and 5IXY (model A) and (ii) alternatively, we developed models using energy-minimized conformations of the initial set of 28 actives that we used in 2D fingerprint screening (model B). Two receptor-based pharmacophore models were developed using PDB ID codes 4ZVV (model C) and 5IXY (model D). We used structures 4ZVV and 5IXY to capture the impact of stereochemistry on ligand binding affinity. The crystal structure 4ZVV is crystallized with the more potent ligand (IC_50_ = 3 nM) in the “R” absolute configuration; the structure 5IXY has an 18-fold less active “S”-isomer bound to the active site.^9,13^

**Figure 3:**
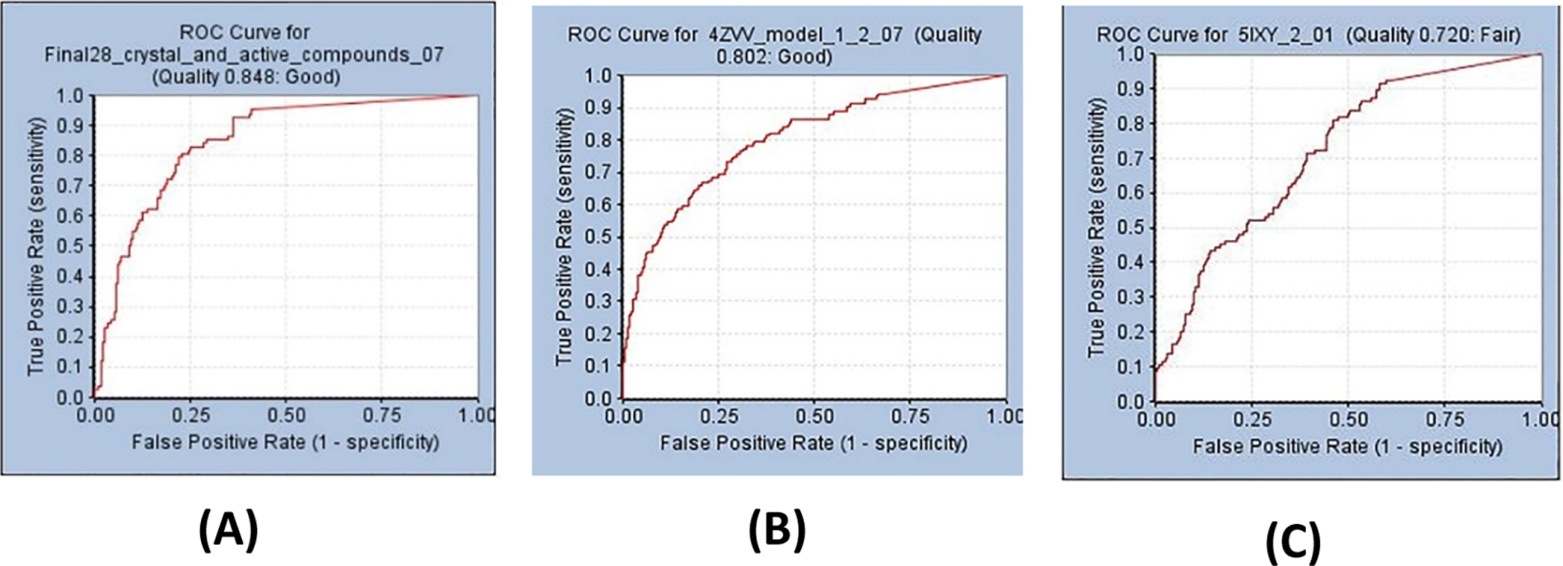
ROC curve from pharmacophore models. (A) Ligand-based model using 28 LDHA (model B) inhibitors. (B) Receptor-based model with X-ray crystal structure PDB ID 4ZVV (model C). (C) Receptor-based model with X-ray crystal structure PDB ID 5IXY (model D).

**Figure 4:**
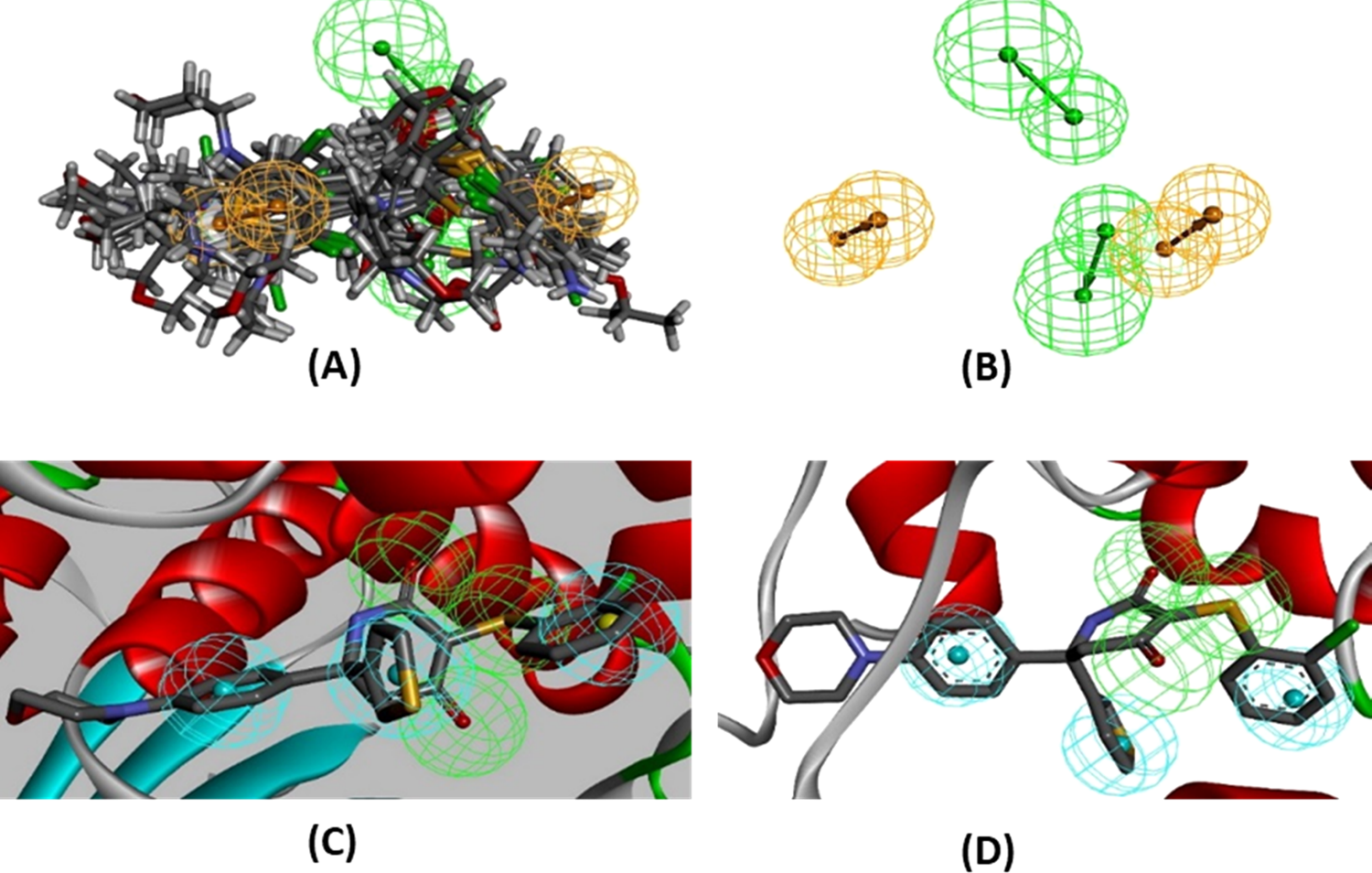
Pharmacophore models of LDHA. (A) Model B (RRAA) built using 28 active LDHA inhibitors. (B) Model B (RRAA) using 28 active LDHA inhibitors shown without ligands. (C) Receptor-based pharmacophore model C (AAAHHH) using the X-ray crystal structure PDB ID 4ZVV. (D) Receptor-based pharmacophore model D (AAAHHH) built using the X-ray crystal structure PDB ID 5IXY.

Out of the four pharmacophore models, the ligand-based model developed with five bioactive ligand conformations (model A) performed poorly. In comparison, the other three models (B, C, and D) gave reasonable hit rates. Model B was obtained using 28 known LDHA inhibitors (Table S5); model C was built with receptor PDB ID 4ZVV (Table S6), and model D with receptor PDB ID 5IXY (Table S7). For database screening, pharmacophore_7, pharmacophore_1, and pharmacophore_1 (Tables S5–S7) were selected as the hypothesis from models B, C, and D, respectively (Table 3). The ROC plots and pharmacophore features from methods B, C, and D are shown in Figures 3 and 4, respectively. The ligand-based model B showed four pharmacophoric features RRAA where R represents an aromatic ring feature, and A means a H-bond acceptor. The receptor-based models C and D showed similar pharmacophoric features, AAAHHH, where A and H represent an H-bond acceptor and hydrophobic feature, respectively. The selected pharmacophores of models B, C, and D were used to screen the 1.14 million compounds identified from 2D screening. As shown in Table 3, both the receptor-based models were more specific and retrieved fewer compounds than the ligand-based model B. A total of 112,549 compounds were obtained from the three models, which were reduced to 84,750 compounds upon removing duplicates.

**Table 3:** Compounds Selected and Advanced from Pharmacophore Models

models	description	hypothesis	number of features	feature set	number of compounds retrieved from models
B	ligand-based model using 28 actives	pharmacophore_07	4	RRAA	56,143
C	receptor-based model using PDB ID 4ZVV	pharmacophore_01	6	AAAHHH	29,674
D	receptor-based model using PDB ID 5IXY	pharmacophore_01	6	AAAHHH	26,732

### Molecular Docking

The 84,750 compounds retrieved from pharmacophore models were prepared by the Ligprep panel in the Schrodinger drug discovery suite, version 2020-2 (Portland, Oregon).^33^ The resulting 184,578 ligprep structures were docked with a three-tier virtual screening workflow protocol in the Glide docking package. In the initial docking step, we used a virtual screening workflow (VSW) using 5IXS as the receptor. VSW involved the molecular docking simulation using high-throughput virtual screening (HTVS), glide standard precision (SP), and glide extra precision (XP) modes. In the HTVS, compounds scored in the top 50% were passed to the next step involving docking with the SP mode in which we selected another top 50% of compounds for docking with glide XP. The resulting 21,188 compounds were docked again in the XP mode using an ensemble docking approach with two more crystal structures with PDB IDs 5IXY and 4ZVV. The top 600 compounds (glide XP score cutoff −7.5 or lower) were selected from structures 5IXS, 5IXY, and 4ZVV. Compounds consistently ranked higher in the ensemble docking approach were prioritized. Binding poses were evaluated, and interactions determined from the WaterMap studies were examined. We selected 40 compounds from screening and purchased and evaluated 29 compounds in LDHA inhibition assays.

### WaterMap Simulations

**Figure 5:**
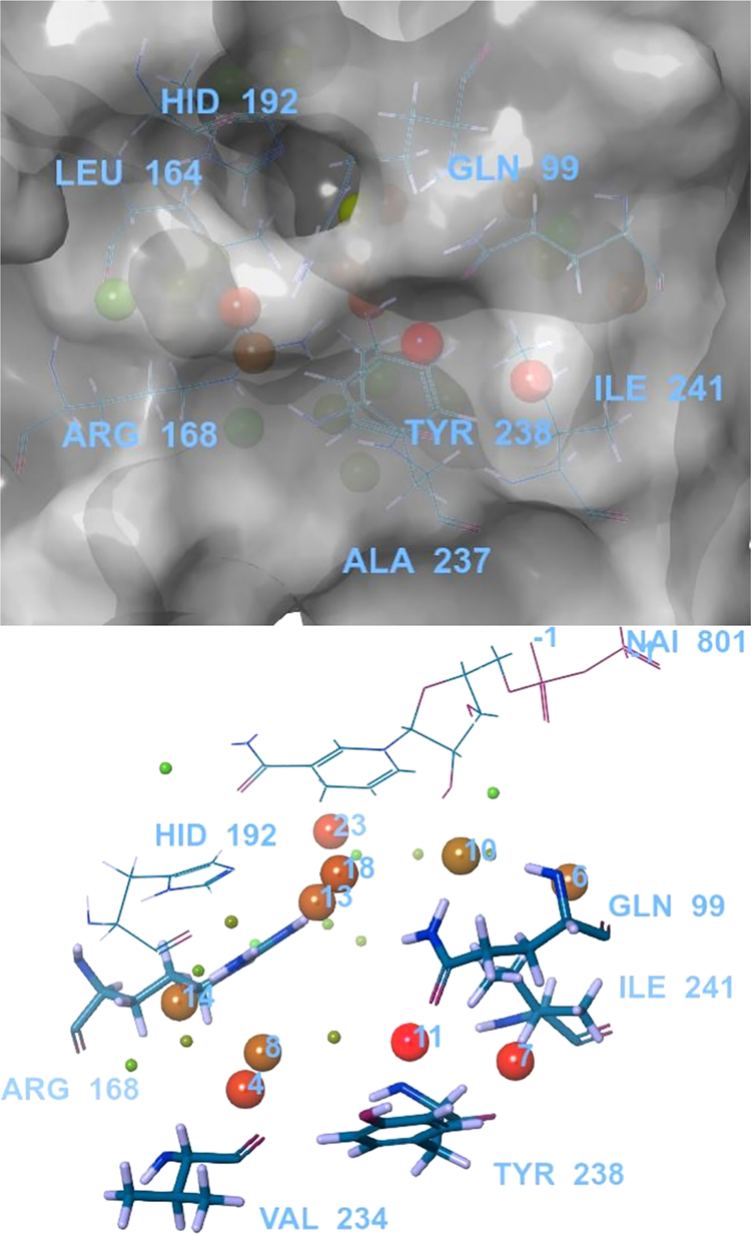
Water sites in the LDHA active site (PDB ID 1I10) adjacent to NADH. The protein is represented as a surface, and amino acids in the LDHA active site are labeled. The hydration sites are numbered. The relatively higher-energy water sites are indicated by red and brown spheres, and green spheres show low-energy water sites.

We studied the effect of thermodynamic properties (entropy, enthalpy, and free energy) of crystallographic water molecules in the LDHA binding site of the oxamate-bound structure, PDB ID 1I10, using WaterMap.^38^ The waters from the simulation are clustered as hydration sites, and the free energy of each water site relative to the bulk solvent (Δ*G*) is calculated by the inhomogeneous solvation theory (IST).^39^ The water site is classified as low or medium energy if they possess Δ*G* ≤ 1.5 or 3.5 kcal/mol, respectively. High-energy water sites or “unstable” waters have Δ*G* ≥ 3.5 kcal/mol.^40^ We evaluated known LDHA inhibitors retrospectively (data not shown) and determined the thermodynamics of the water sites (Table 4). The WaterMap on the LDHA active site and the key amino acids that could be targeted are illustrated in Figure 5. Most unstable hydration sites include sites 11 and 7, close to Tyr238, Ile241, and Gln99. Another unfavorable hydration (site 4) was adjacent to Arg168, while sites 18 and 13 were close to His192.

**Table 4:** Thermodynamic Analysis of Water Molecules at the LDHA Active Site

hydration site	Δ*G*	Δ*H*	–*T*Δ*S*
11	10.26	6.05	4.21
7	6.98	2.57	4.41
4	6.19	0.72	5.47
23	6.12	5.04	1.08
18	5.19	1.24	3.95
13	4.87	1.09	3.78
6	4.52	–0.61	5.13
8	4.46	0.18	4.28
14	4.30	0.15	4.15
10	3.83	–1.07	4.90

### Inhibition of LDHA

**Figure 6:**
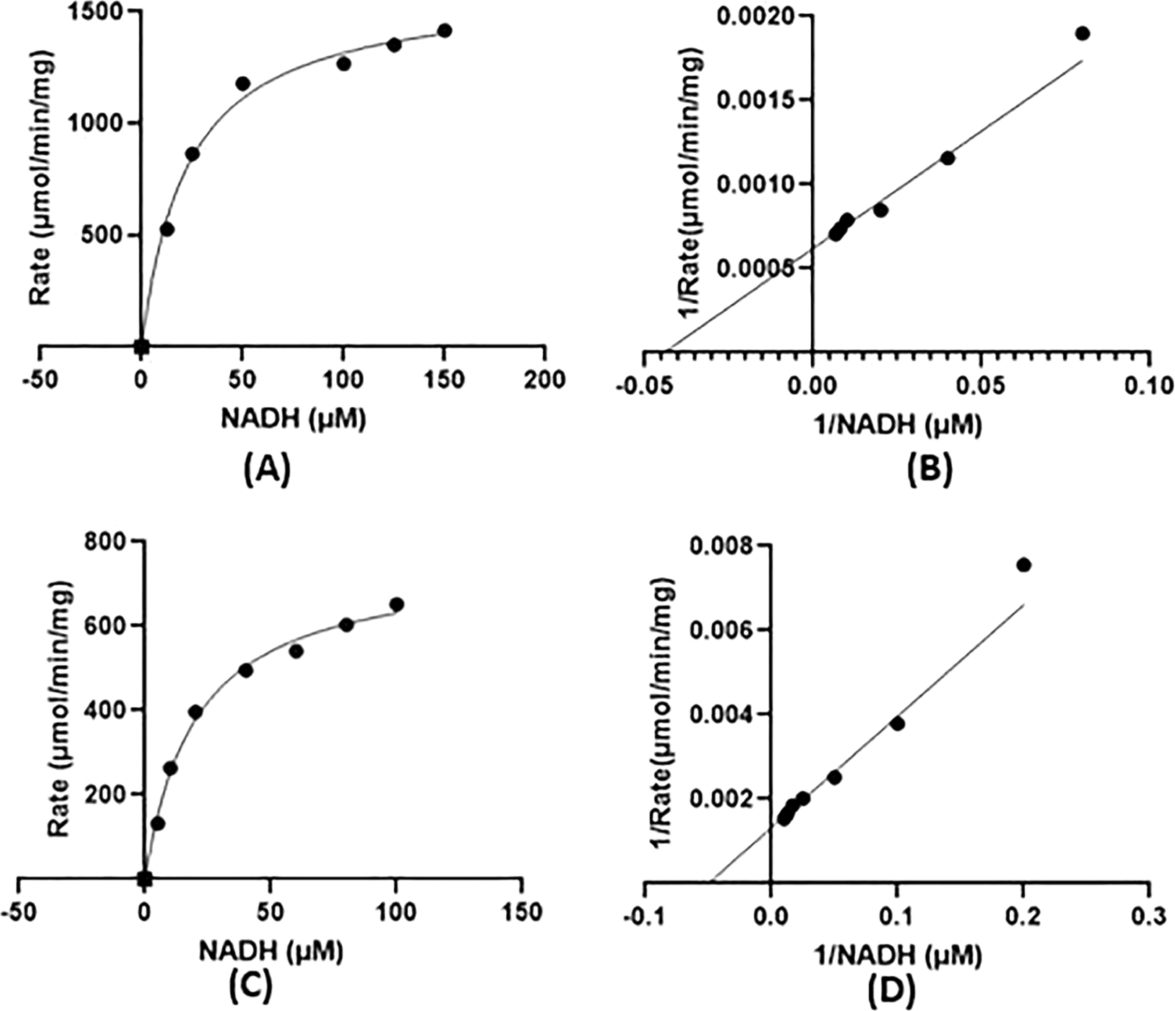
Enzyme kinetics. (A) Michaelis–Menten curve, and (B) corresponding lineweaver Burk plot for hLDH5 (LDHA); (C) Michaelis–Menten and (D) lineweaver Burk plot for hLDH1 (LDHB).

Michaelis–Menten constant for NADH was determined from the initial rate measurements at 37 °C using a nonlinear regression analysis and is represented by a lineweaver Burk plot (Figure 6). For hLDH5 (LDHA), in agreement with the literature, NADH showed an average *K*_m_ of 19.4 μM and an average *V*_max_ of 1629.3 μmol/min/mg.^41^ For hLDH1 (LDHB), NADH showed an average *K*_m_ of 18.96 μM and an average *V*_max_ of 700.6 μmol/min/mg. Twenty-nine compounds selected from virtual screening were evaluated in LDHA inhibition assays. Six compounds (Figure S2) contain the succinic acid monoamide moiety with substitution at the alpha-carbon adjacent to the carboxylic acid. Compound **ZINC1162757** has a substitution on the beta carbon, while **ZINC10287535** does not have a substitution at either position. Other compounds have substitutions at the beta carbon but with a reverse amide group (Figure S3). Another group of compounds has a different hydroxy pyrimidinone ring system (Figure S4). Some structurally more diverse compounds selected for biological evaluation are shown in Figure S5. Carboxylic acid or other acidic hydrogen is a common feature of most known LDHA inhibitors (Figure 1) and could be essential for activity. Interestingly, most compounds selected for screening contain a carboxylic acid moiety or a weakly acidic enolic group of the hydroxy pyrimidinone ring.

**Figure 7:**
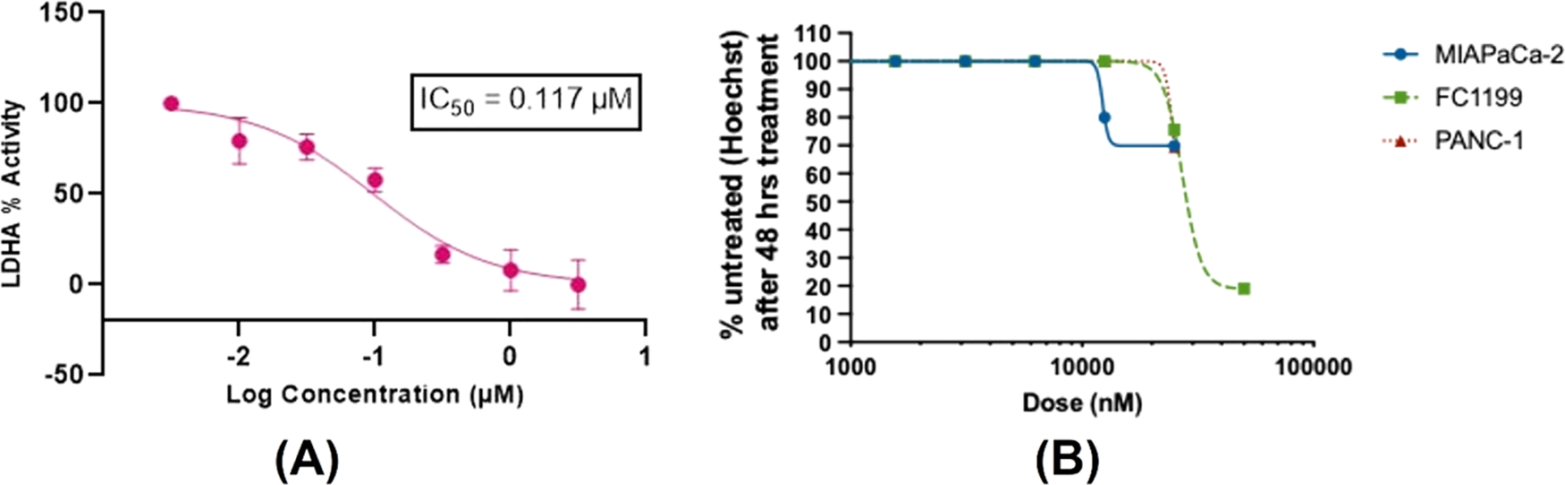
Dose–response curve of ZINC13469319. (A) Biochemical LDHA inhibition assay. (B) Cell viability assay in pancreatic cancer cell lines. The assays were performed in triplicates and the data is presented as mean ± SD (*n* = 3).

Five–eight scalar concentrations of compounds were prepared to develop a dose–response curve. A full dose–response curve for compounds showing no or minimal inhibition at 100 μM concentration was not determined, and their IC_50_ is reported to be >100 μM (Table 5). Compounds with substitutions at alpha- or beta-positions on the succinic acid monoamide moiety are tolerated, while the compound without substitution, **ZINC10287535**, did not exhibit activity. Four compounds containing a reverse amide showed moderate inhibition of LDHA, suggesting that structural optimization and SAR studies may improve their potency. **ZINC13469319** showed an IC_50_ of 117 nM, the most potent compound identified from screening (Figure 7). Further, most compounds containing a hydroxy pyrimidinone ring showed an activity with IC_50_ in single-digit and low micromolar concentrations. Interestingly, compounds with hydroxy pyrimidinone rings have some structural similarity to the known potent LDHA inhibitor **GNE-140**, which possesses a hydroxy lactam ring. **ZINC2783354** represents a promising hit with IC_50_ = 9.45 μM. The binding free energies (Δ*G*_bind_) of **ZINC13469319** and **ZINC2783354** from the MM-GBSA correlated with the experimental affinity as reflected with Δ*G*_bind_ values of −57.55 and −21.0 kcal/mol, respectively. Although the role of LDHB is not very well understood in cancer, it is likely it could contribute to metabolic plasticity. We evaluated representative hits in the LDHB inhibition assay. **ZINC13469319** and **ZINC2783354** showed IC_50_ values of 0.254 and 56.72 μM, suggesting they are more selective at inhibiting LDHA. In our assay, **NHI-2** showed IC_50_ ∼ 21.0 μM, which is comparable to its reported value (IC_50_ ∼ 15.0 μM).^10^

**Table 5:** Biochemical Activity of Compounds Identified from Virtual Screening

compound	LDHA (IC_50_ ± standard deviation (SD))(μM)^a^
**ZINC13469319**	0.117 ± 0.0287
**ZINC16482404**	55.74 ± 1.29
**ZINC4978206**	21.92 ± 2.24
**ZINC2783354**	9.45 ± 1.83
**ZINC4686101**	40.65 ± 8.54
**ZINC13225109**	21.19 ± 1.59
**ZINC10059010**	9.74 ± 3.21
**ZINC2745830**	55.82 ± 8.59
**ZINC95424079**	60.09 ± 7.60
**ZINC25204967**	62.35 ± 4.72
**ZINC12817529**	24.37 ± 6.44
**ZINC8575365**	62.93 ± 2.46
**ZINC8579113**	27.76 ± 3.17
**ZINC4580599**	48.42 ± 5.91
**ZINC69492082**	14.08 ± 2.23
**ZINC6278574**	13.90 ± 6.88
**ZINC1162757**	16.31 ± 4.72
**ZINC3626961**	>100
**ZINC83975796**	>100
**ZINC10287535**	>100
**ZINC13224346**	>100
**ZINC58848041**	>100
**ZINC13176721**	>100
**ZINC16481336**	>100
**ZINC55151005**	>100
**ZINC72260234**	>100
**ZINC620615**	>100
**ZINC40267182**	>100
**ZINC61718959**	>100
**NHI-2**	21.32 ± 0.617

a The assays were performed in triplicates and the data is presented as mean ± SD (*n* = 3).

### Cytotoxicity against Pancreatic Cancer Cell Lines

**Figure 8:**
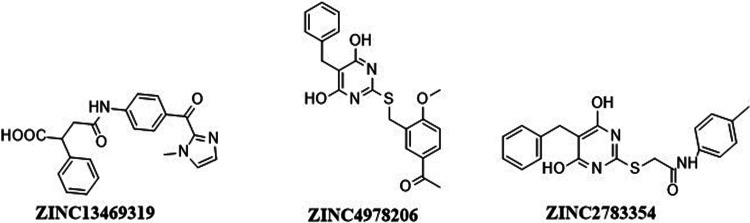
Structures of potent hits selected for cytotoxicity studies.

Three hits shown in Figure 8 were selected and tested against human pancreatic cancer PANC-1 and MIAPaCa-2 cells, obtained from the American Type Culture Collection (ATCC, Manassas, Virginia). Compounds were also tested against mouse pancreatic cancer FC1199 cells from the KPC-1 genetically engineered mouse model that has mutated K-Ras, metastasized to the liver, and formed desmoplasia (Table 6). The assay was carried out under normoxic conditions. Anticancer potency was investigated using a fluorescence-based high-throughput confocal microscopy cell viability/proliferation/cell death assay.^42^ To rule out the nonspecific cytotoxicity, we tested compounds in hTERT immortalized normal pancreatic (HPNE) cell lines. **ZINC2783354** containing the hydroxy pyrimidinone ring inhibits PANC-1 and MIAPaCa-2 viability with IC_50_ = 14.64 and 25.85 μM, respectively.

**Table 6:** Cytotoxicity of Selected Hits

compound	PANC-1IC_50_ ± SD (μM)	MIAPaCa-2IC_50_ ± SD (μM)	FC1199 IC_50_ ± SD (μM)	HPNE normal IC_50_ ± SD (μM)
**ZINC4978206**	24.996 ± 1.523	14.269 ± 5.012	50.630 ± 3.104	25.118 ± 4.200
**ZINC2783354**	14.640 ± 0.890	25.851 ± 8.212	12.613 ± 1.292	>50.0
**ZINC13469319**	24.75 ± 2.28	12.26 ± 1.12	27.09 ± 3.06	>50

Further, **ZINC2783354** does not display toxicity toward normal cells (IC_50_ > 50 μM). **ZINC13469319** containing the succinic acid monoamide moiety showed greater potency against MIAPaCa-2 cells with cell viability IC_50_ = 12.26 μM. **ZINC13469319** showed no apparent cytotoxicity against the normal pancreatic duct cell line hTERT-HPNE. The morphological images of PANC-1, MIAPaCa-2, and FC1199 cells upon treatment with **ZINC13469319** are shown in Figures S6–S8, respectively. It suggests that the cell-killing mechanism could be due to early apoptosis within probably 24 h, followed by necrosis as indicated by disruption of the plasma membrane. Ongoing studies in our lab will characterize the cell-killing mechanism of these compounds in more detail. Relative to the biochemical potency of **ZINC13469319** (IC_50_ = 0.117 μM), the drop in cellular activity (MIAPaCa-2 IC_50_ = 12.26 μM) could be due to the presence of a carboxylic acid that can limit its penetration into the cells. **ZINC2783354**, which possesses a weakly acidic enolic group, has comparable biochemical (IC_50_ = 9.45 μM) and cell viability (PANC-1 IC_50_ = 14.64 μM). Both hits are similar or more potent than **NHI-2**, inhibiting PANC-1 cell growth with a reported IC_50_ value of 22.2 μM.^12^ Interestingly, the cytotoxicity of these hits, under normoxic conditions, against pancreatic cancer cell lines is comparable to one of the most potent LDHA inhibitors, **GNE-140**, which inhibits PANC-1 with an IC_50_ value of 11.93 μM under normoxic conditions and is more potent under hypoxic conditions with IC_50_ = 2.05 μM against MIAPaCa-2.^13,43^

### Inhibition of Lactate Production

**Figure 9:**
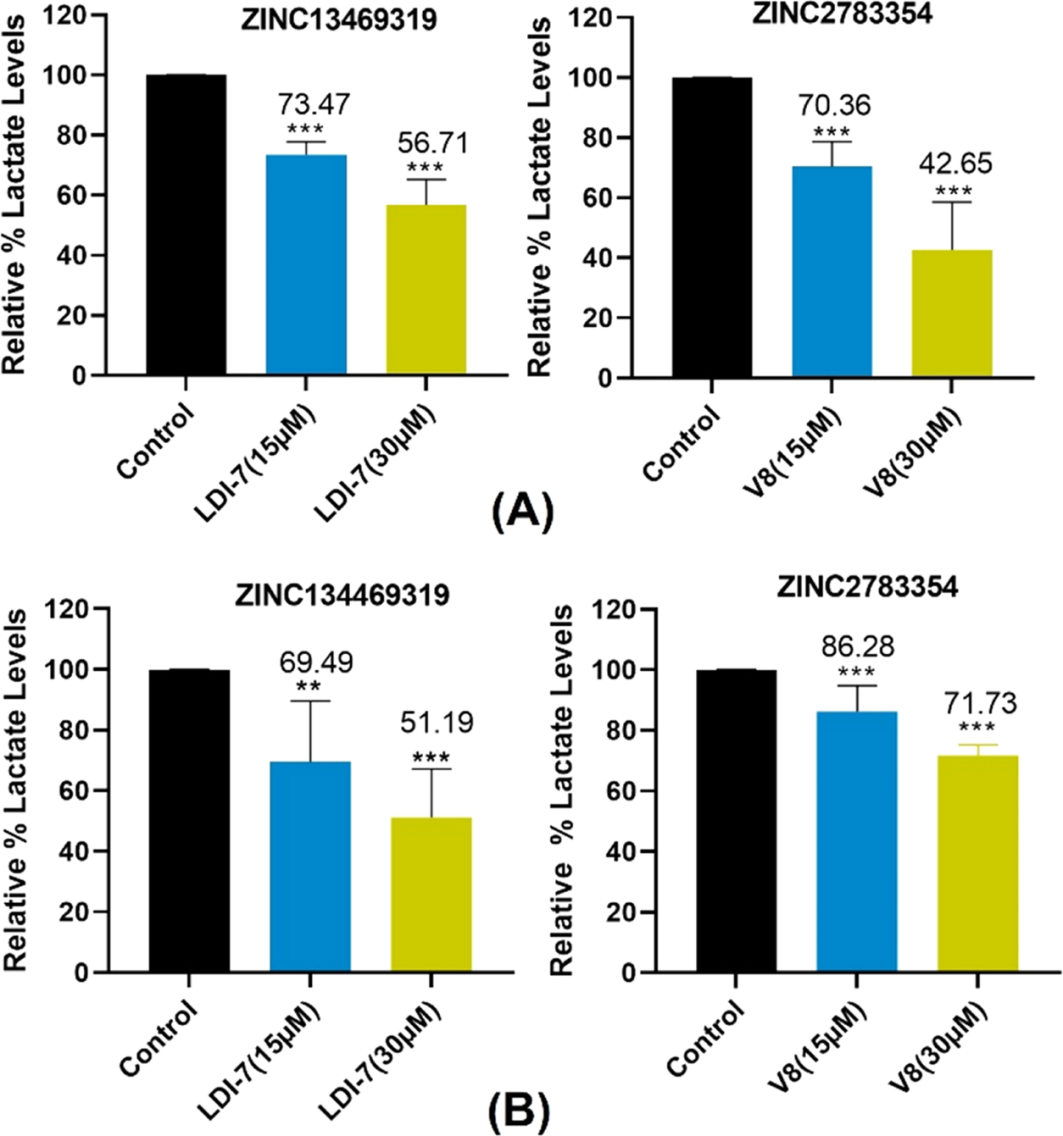
Inhibition of lactate production by **ZINC13469319** and **ZINC2783354** in MIA PaCa-2 cells in (A) cell culture medium containing 10 mM glucose, 2 mM glutamine, and without pyruvate. (B) Cell culture medium containing 25 mM glucose, 2 mM glutamine, and 1 mM pyruvate. Lactate levels are reported as mean ± SD. Data were statistically compared using one-way analysis of variance (ANOVA). The experiment was repeated 3–4 times. **P ≤ 0.01, ***P ≤ 0.001. *P* < 0.05 is considered statistically significant.

Lactate production is regarded as a critical event involved in carcinogenesis and immune escape.^6,44,45^ MIAPaCa-2 cells were treated with **ZINC13469319** and **ZINC2783354**, and lactate accumulated in the cell culture was determined after 6 h.^13,46^Figure 9 shows that both compounds demonstrated inhibition of lactate production in a dose-dependent manner. Compounds were tested in varied nutrient conditions. Initially, we used the regular DMEM medium (25 mM glucose, 2 mM glutamine, and 1 mM pyruvate). Next, we evaluated compounds in nutrient-stressful conditions with low glucose (10 mM) and no pyruvate in the cell culture media. **ZINC13469319** and **ZINC2783354** resulted in ∼30 to 50% reduction in lactate levels when treated with 15 and 30 μM concentrations, respectively.

### Molecular Dynamics

**Figure 10:**
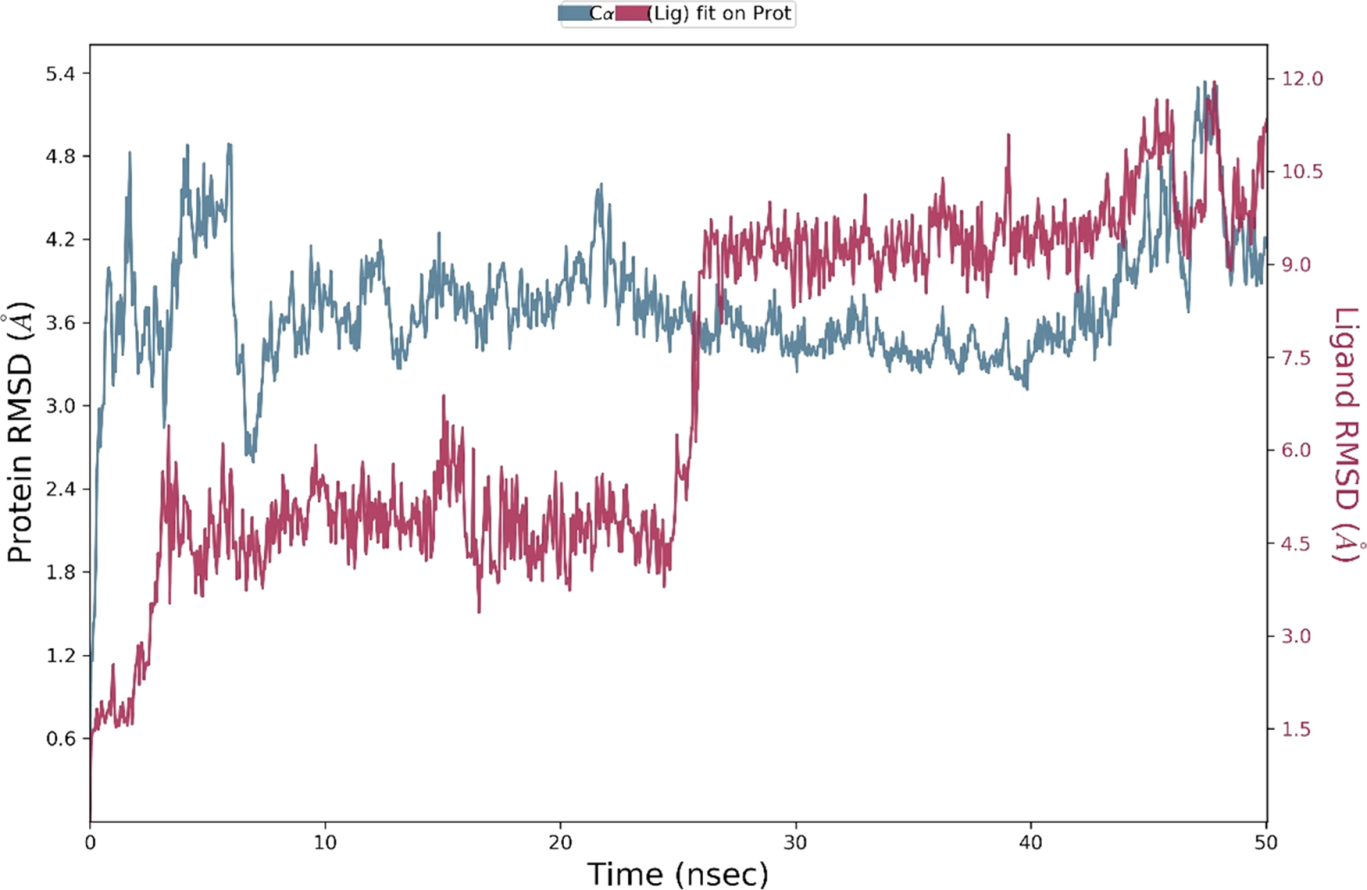
Protein (LDHA)–ligand (**ZINC13469319**) (PL) RMSD from the 50 ns MD simulation. Blue indicates RMSD of Cα backbone atoms on the protein over the 50 ns simulation. Blue indicated ligand RMSD on the protein over the 50 ns simulation.

**Figure 11:**
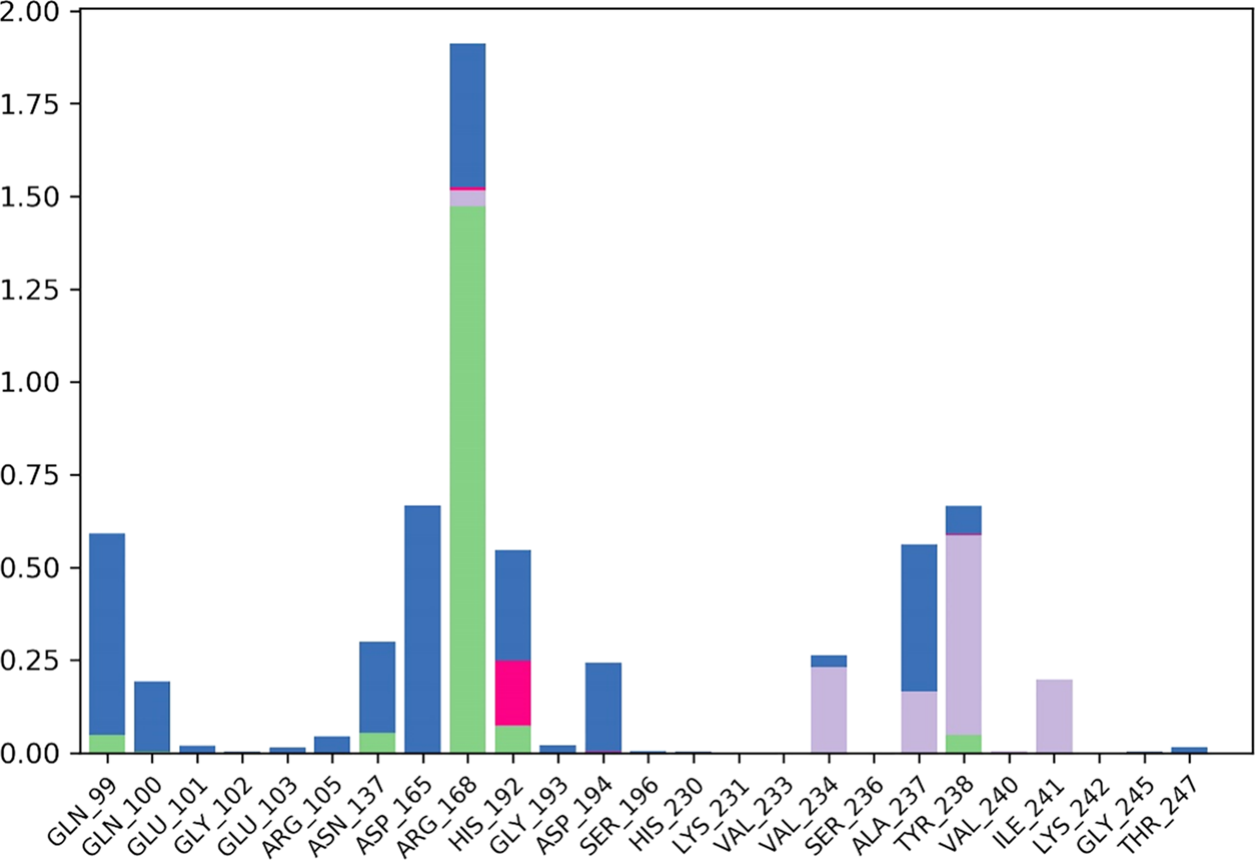
Protein (LDHA)–Ligand (**ZINC13469319**) contact histogram from the 50 ns MD simulation.

**Figure 12:**
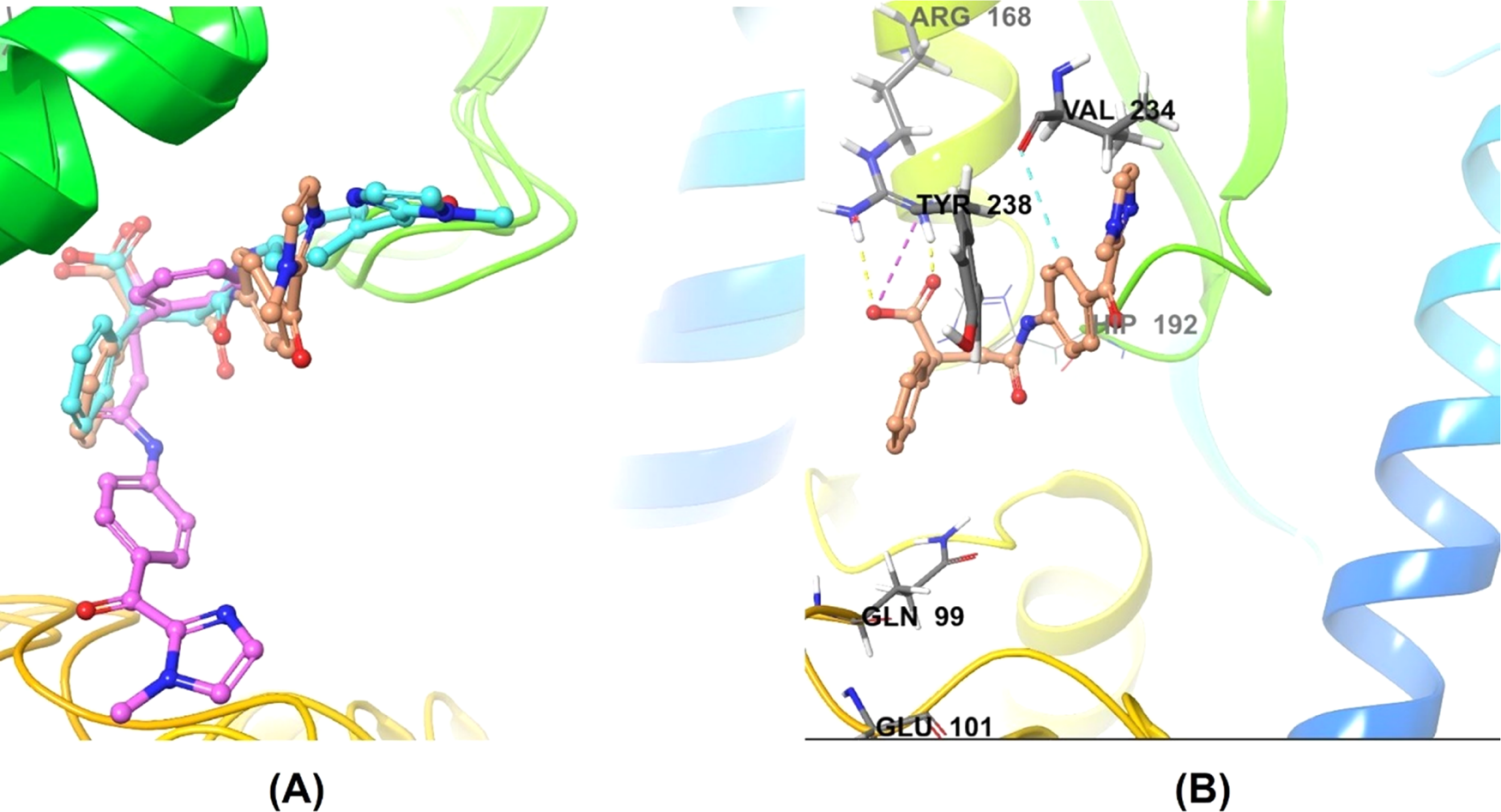
(A) Three representative poses of **ZINC13469319** from clustering MD trajectories. (B) Representative pose (shown by the ligand with brown carbons) attained at the equilibrium shows the interaction with Tyr238 and Val234. The cofactor, hydrogens, and water molecules are not displayed for clarity.

**Figure 13:**
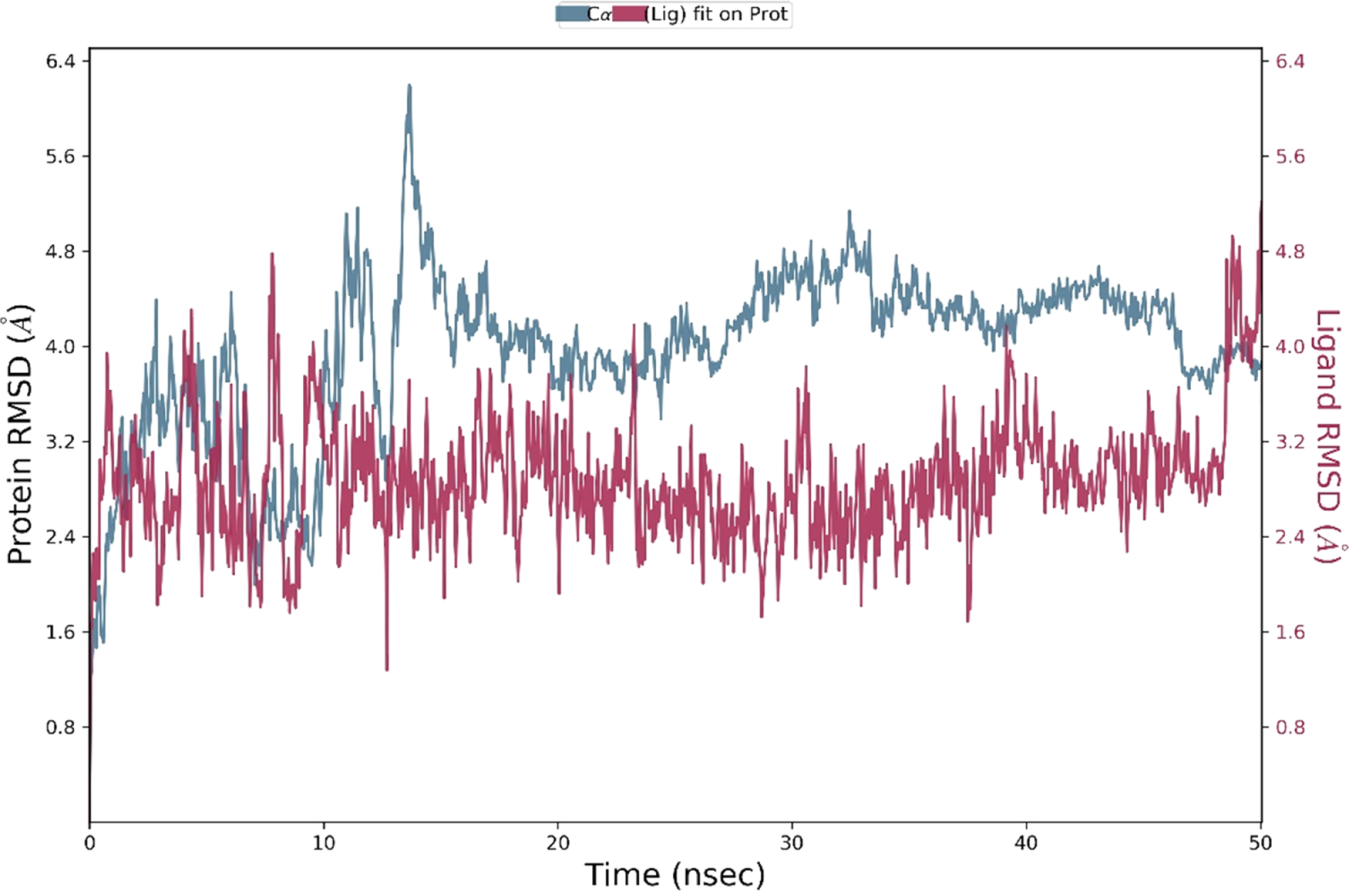
MD simulation of **ZINC2783354** showing the protein–ligand (PL) RMSD. Blue indicates RMSD of Cα backbone atoms on protein (LDHA) over the 50 ns simulation. Blue indicated ligand RMSD on the protein over the 50 ns simulation.

**Figure 14:**
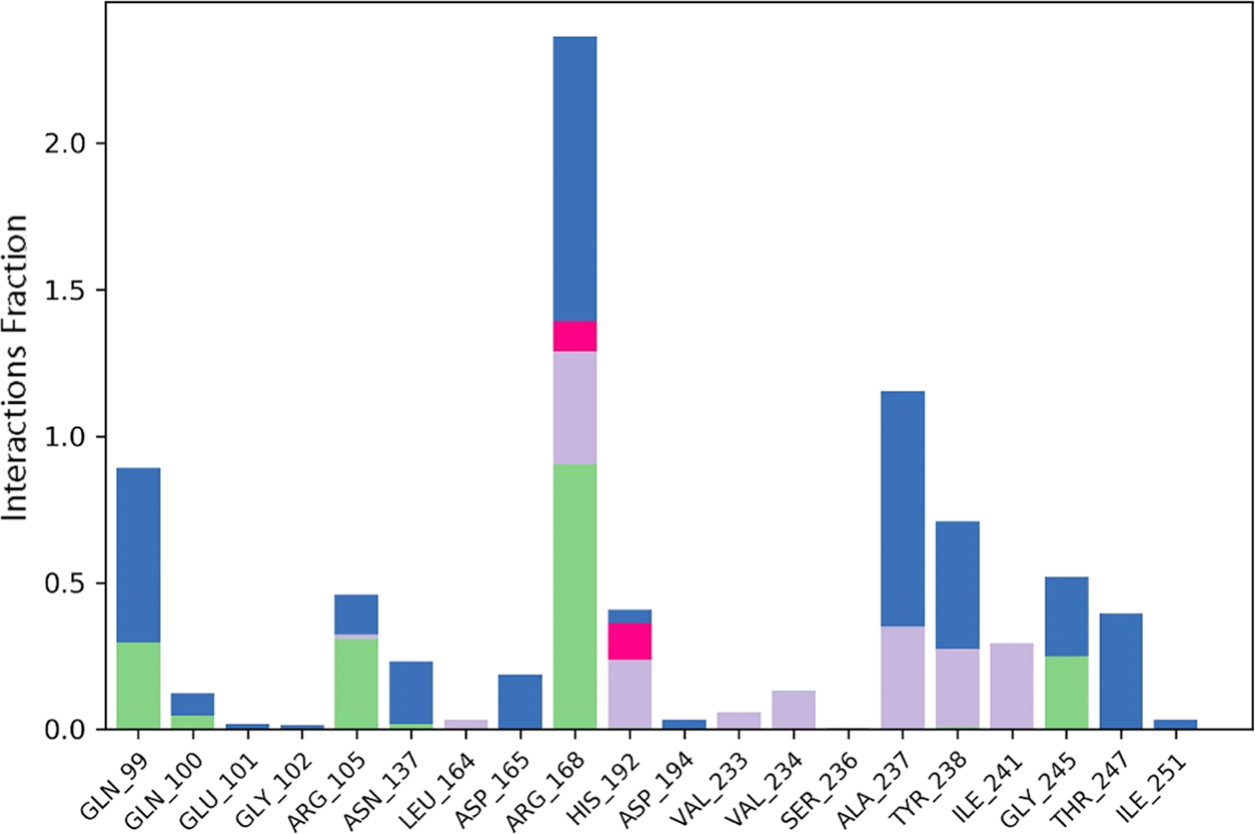
Protein (LDHA)–ligand (**ZINC2783354**) (PL) contact histogram from the 50 ns MD simulation.

**Figure 15:**
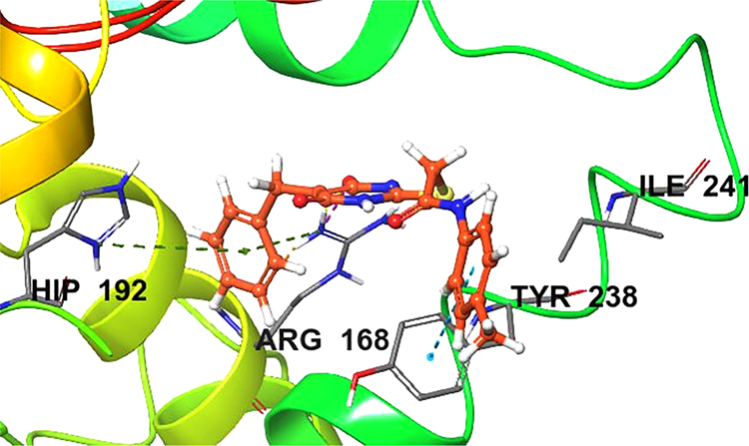
Representative pose of **ZINC2783354** from MD trajectories. Interactions with key amino acids are shown.

The interactions of the two most potent hits, **ZINC13469319** and **ZINC2783354**, were further studied by 50 ns MD simulations of the protein–ligand complexes.^47^ To understand the stability of the complex during the MD simulation, the protein backbone frames were aligned to the backbone of the initial frame. Protein–ligand (PL) RMSD, PL contact histogram, and protein secondary structure element (SSE) were analyzed to check the stability, fluctuations, and PL contacts during the simulation. The protein–ligand RMSD plot of **ZINC13469319** and the PL contact histogram are shown in Figures 10 and 11. The ligand showed fluctuations in the initial 7 ns of the simulation, followed by which equilibrium was reached, and the ligand remained stable for the remaining time of the simulation. To capture the representative snapshots that evolved, we used the Desmond Trajectory Clustering panel in the Schrödinger suite and separated the frames from each trajectory in three clusters based on the RMSD of Cα atoms of the protein backbone. The cluster representing the snapshot obtained upon stabilization had a greater number of members, 15, compared to other groups that had 8 and 5 members, and a more favorable MM-GBSA Δ*G*_bind_ (−48.83, −41.30, and −36.72 kcal/mol) when compared to the snapshots from the other clusters. The superimposed ligand conformations from the three clusters and the representative conformation evolved over binding along with protein–ligand contacts are shown in Figure 12. In the initial few seconds of simulation, the ligand’s phenone and the imidazole ring are directed toward the loop residues Gln99, Gln100, and Glu101. Upon stabilization, the phenone and imidazole ring flipped and were observed close to Tyr238 and Val234. In this conformation (panel B, Figure 12), the carboxylic acid group of the ligand made H-bonding and salt bridge interactions with Arg168. Other interactions with amino acids, including Tyr238, Ala 237, His192, Asp194, Asn137, and Val234, were observed within 5 Å distance. The protein–ligand RMSD plot of **ZINC2783354** (Figure 13) shows the ligand was stable for the entire 50 ns simulation time. The PL contact histogram and a representative snapshot of the ligand are shown in Figures 14 and 15, respectively. The enolic group of the hydroxy pyrimidinone ring interacts with Arg168. The phenyl of the benzyl group makes cation−π interaction with His192, while the methyl-substituted phenyl ring made contact with Tyr238 and Ile241.

## Conclusions

LDHA is a promising anticancer target for cancer therapy, including PDAC. Currently, no LDHA inhibitors are in clinical development partly due to limitations associated with their molecular structures, which either lack drug-like properties or have poor pharmacokinetics. Thus, efficient exploration of greater chemical diversity is urgently required. We employed a screening strategy that searched a virtual library of 15 million compounds and identified several chemotypes as novel LDHA inhibitors. Compounds cause a 30–50% reduction in lactate production in MIAPaCa-2 cells when tested at 15 and 30 μM concentrations.

Further, selected hits inhibit the viability of several pancreatic cancer cell lines. Generally, compounds identified belong to two chemical classes and contain a succinic acid monoamide group or a hydroxy pyrimidinone ring. In the succinic acid series, the most potent hit, **ZINC13469319**, inhibited LDHA with IC_50_ of 117 nM and demonstrated cytotoxicity against MIAPaCa-2 with IC_50_ = 12.26 μM. Among the hydroxy pyrimidinone series, **ZINC2783354** has comparable biochemical (IC_50_ = 9.4 μM) and cytotoxic (PANC-1 IC_50_ = 14.26 μM) potency. Both hits were selective for cancer cells and did not cause any apparent cytotoxicity to normal cells. Synthesis and structure–activity relationship of hits identified will be reported in subsequent papers.

## Experimental Section

### Pharmacophore Modeling

We used the Catalyst Pharmacophore Modeling and Analysis toolset implemented in BIOVIA Discovery Studio (Accelrys Software, Inc., San Diego, California).^48^ We used the FAST conformational analysis method for conformer generation. The maximum number of conformers generated for each molecule was set to 255 with an energy threshold of 20 kcal/mol above the estimated global minimum energy. The FAST protocol of catalyst is based on the hypothesis that the low-energy conformational spaces of small molecules can be adequately sampled by a small collection of conformations, which can effectively represent a more extensive quasi-exhaustive set of conformers.^49^ The fast approach applies a modified systematic search. Next, the conformations are energy-minimized in a restricted CHARMM force field to generate conformations within the defined energy cutoff. Finally, heuristics ensure that the subset of conformations, limited by the maximum number of outputs, has full conformational diversity. Common features selected for screening include hydrogen bond donor (D), hydrogen bond acceptor (A), hydrophobic group (H), ring aromatic (R), and positive (P) and negative (N) ionizable groups. The pharmacophore was assigned a minimum of 4 and a maximum of 10 features. Ten hypotheses were generated, with the minimum interfeature distance set to 2.97.

### Molecular Docking

The Glide molecular docking package of Schrödinger was used for docking.^33^ We used the protein preparation wizard of the Maestro (v) interface in the Schrödinger modeling package to prepare the protein. The compounds for docking were prepared using the LigPrep module in the Schrödinger modeling package. Protein crystal structures were prepared using the protein preparation wizard. Hydrogens were added, bond orders were assigned, and the missing side chains and loops were added using the Prime package in Schrödinger. The hydrogen bonding network was optimized by reorienting the hydroxyl and thiol groups and amide groups of Asn, Gln, and His side chains. Neutral and protonated states of His, Asp, and Glu and tautomeric states of His were sampled at pH 7.0 using PROPKA. Following H-bond optimization, the protein was minimized using the OPLS-2005 force field until the RMSD of heavy atoms converged to 0.30 Å. The receptor grid was constructed with NADH, and the docking site was set to the centroid of the workspace ligand, with one positional and one H-bonding constraint. Ligands with a length up to 20 Å were allowed to dock. To ensure glide reproduces bioactive ligand conformations, we evaluated glide’s standard precision (SP) and extra precision modes (XP). RMSDs between all heavy atoms obtained upon overlay of docked and bioactive ligand conformations of ligands from structures 5IXS, 4R69, 4RLS, and 4ZVV range from 0.07 to 0.80 Å for SP and 0.07–0.75 Å for XP mode. It suggested that both SP and XP modes successfully reproduced bioactive X-ray ligand conformations of the target of interest. We used glide XP for pose generation, and the docking was terminated if two consecutive solutions were within an RMSD of 0.5 Å.

### WaterMap Calculations

We used the WaterMap program of Schrödinger to determine the free energy, enthalpy, and entropy of surface water molecules in the LDHA active site.^38^ The structure of LDHA (PDB ID 1I10) was prepared using the protein preparation wizard. The crystallographic waters were retained for the calculations. The binding site was defined by the ligand oxamate, and waters within 10 Å of the selected atoms were selected for analysis. Both the “apo” and “holo” WaterMaps were prepared. The “truncate protein” option was unchecked in the simulation setup, and all existing waters were treated as the solvent. The 2.0 ns MD simulations were carried out using the OPLS4 force field on an NVIDIA A100 GPU cluster.

### LDH Inhibition Assay

The hLDH5 (LDHA) inhibition assay was performed by measuring the fluorescence (excitation@340 nm, emission@460 nm) and monitoring the NADH conversion rate to NAD^+^ at 37 °C as reported.^10^ The apparent Michaelis–Menten constant (*K*_m_) of NADH for hLDH5 was determined using 0.003 units of hLDH5 per well under saturated pyruvate (1440 μM) and increasing NADH concentrations from 12.5 to 150 μM in 100 mM sodium phosphate buffer (pH 7.4). Michaelis–Menten constants were determined with nonlinear regression analysis using GraphPad Prism 9.0. The above conditions were used for the hLDH5 inhibition assays, which were carried out in 96-well plates with the following final enzyme and buffer concentration: 100 mM phosphate buffer (pH = 7.4), 0.003 units of LDHA, 40 μM NADH (∼2 × *K*_m_), and 1.44 mM pyruvate (saturated pyruvate conditions). The stock solution of compounds was prepared in dimethyl sulfoxide (DMSO). **NHI-2** and DMSO were used as positive and negative controls, respectively. The experiment involved adding a solution of compounds in DMSO to the enzyme and NADH in a phosphate buffer. The assay plate was incubated at 25 °C for 10 min, and a baseline read was taken, after which pyruvate was added. The fluorescence was read for 5 min every 30 s in a microplate reader. The slope of a suitable linear timeframe was calculated with the curve bottom assigned to the initial 5-s recording before adding pyruvate (background rate) and the curve top to the negative (DMSO only) control wells rate. IC_50_ values were determined from dose–response curves using the four-parameter logistic nonlinear regression analysis in Prism Software v9.0. The assays were performed in triplicates and the data is presented as mean ± SD (*n* = 3). The *K*_m_ for hLDH1 was determined with 0.0026 units of hLDH1 using saturated pyruvate and increasing NADH concentrations (5–100 μM). The hLDH1 inhibitory activity of promising hits was determined using similar assay conditions with 0.0026 units of hLDH1, 1440 μM pyruvate, and 40 μM NADH (∼2 × *K*_m_) concentrations.

### Cell Lines

PANC-1, MiaPaCa-2, and HPNE cell lines were obtained from American Type Culture Collection (ATCC, Manassas, Virginia). FC1199 cells were obtained from Dr. David Tuveson, Cold Spring Harbor Laboratory. All cell lines were cultured in DMEM (Hyclone, DMEM/high glucose, Cat# SH30243.01 with the addition of 10% Cosmic Calf Serum) (GIBCO, DMEM/high glucose, DMEM Cat# 11995040 with 10% FBS) and Pen/Strep and maintained in a 37 °C, 5% CO_2_/95% humidified air incubator.

### Cytotoxicity Assay

Cytotoxicity was assessed using the viability/proliferation/necrosis assay. For this assay, cancer cells were plated on a 96-well plate, and 1:2 serial dilution of compounds was done with 25 μM as the maximum dose for 2 h in serum-free media, and then 10% serum was added for an additional 46 h. Compounds were prepared in DMSO, which did not exceed a maximum concentration of 0.2%. At the end of 48 h period, Hoechst 33342 (1.0 μM) and SYTOX Green (0.5 μM) fluorescent dyes were added to each well for 15 min. Confocal images were acquired using the Operetta High Content Imaging System (PerkinElmer). In each well, five fields were screened using a 10× field objective with Hoechst 33342 detected using an excitation wavelength of 360–400 nm and an emission wavelength of 490–500 nm. SYTOX Green was detected using an excitation wavelength of 500–520 nm and an emission wavelength of 520–530 nm. Bright-field images were acquired for each field. Images were analyzed using Harmony software (PerkinElmer) with the cell-permeable vital dye Hoechst 33342 to identify cell nuclei (i.e., for cell counts), while the normally cell impermeable SYTOX Green was be used to identify necrotic cells. Cell counts were summed over the five fields for each well, and the percentage of viable cells was calculated relative to the untreated control. Linear regression dose–response (variable slope) analysis was used to calculate the concentration at which the drugs induce 50% cell death, an IC_50_ value, for each extract using Prism Software version 9.0. Standard deviations are reported for IC_50_ values representing >3 biological replicates (3 technical replicates/biological replicates) per compound.

### Lactate Accumulation Assay

Lactate production in the medium was detected using the Lactate Assay Kit (catalog # K-607, BioVision, Mountain View, California). Specifically, an equal number of MIAPaCa-2 cells (4 × 10^5^/well) were seeded in standard DMEM growth medium in a 6-well Nunclon plate and permitted to adhere overnight. The next day, the cells were washed with PBS and treated with compound or vehicle control (v/v%) for 6 h in a treatment media comprising DMEM with varied glucose and pyruvate concentrations and without serum and phenol red. At the end of 6 h, 2 μL of culture medium was taken for the lactate assay and diluted 100-fold with the lactate assay buffer. The cells were collected and lysed, and the lysate was used for protein quantification. The lactic acid secreted in the medium was determined per the manufacturer’s protocol with fluorescence measured at *E*_x_/*E*_m_ = 535/587 nm. A standard curve was used to quantitate the lactic acid in the culture medium. The results were normalized based on the total protein. Experiments were performed in triplicate and repeated at least three times. The data were normalized to untreated cells (control), and the percent lactate production was calculated as (lactate in the control wells – lactate in the experimental group)/control group × 100%. Statistical analysis was conducted using Prism 9.0 software (GraphPad). Differences between the groups were explored by one-way analysis of variance (ANOVA) followed by Dunnett’s multiple comparison test. *P* value < 0.05 was considered significant.

### Protein Extraction

After 6 h of treatment with the inhibitors, the cells were gently scrapped and lysed using ice-cold RIPA buffer supplemented with protease inhibitors. The lysate was incubated (4 °C for 20 min) and then centrifuged (15,000*g*, 20 min, 4 °C) to collect the supernatant containing the total proteins. The total protein in cell lysates was then quantified using the BCA assay kit (catalog#23227).

### Molecular Dynamics

Desmond package in Schrödinger (version 2020-1) was used for the MD simulation on the NVIDIA A100 Tensor Core GPU cluster. The System Builder panel was used to define the solvent and the boundary conditions. TIP3P was used as the explicit solvent model, a boundary condition with an orthorhombic water box of 20 × 20 × 20 Å^3^ buffer region between the ligand atoms and the simulation box boundary, and a 639,957 Å^3^ minimized volume of the box was applied. The net charge of the solvated system was neutralized with Na^+^ counterions, and the salt concentration was set to 0.15 M. The solvated box was energy-minimized using the OPLS4 force field. A 50 ns MD simulation was performed using a 10 ps recording interval, resulting in 1000 frames. An NPT ensemble (isothermal–isobaric ensemble, constant temperature, constant pressure, constant number of particles) using a Nose–Hoover chain thermostat at a temperature of 300 K and relaxation time of 1.0 ps and Martyna–Tobias–Klein Barostat with a pressure of 1 bar and relaxation time of 2.0 ps was applied. The integration time step was set to 2 fs, and for Coulombic interactions, a cutoff radius of 9.0 Å was applied.

### MM-GBSA Calculations

The MM-GBSA binding free-energy calculations were done with the Prime package in Schrödinger, using the VSGB solvation model, and the energies were calculated using the OPLS4 force field. Residues within 5 Å of the ligand were treated as flexible.

## Data Availability

The PDB files were obtained from the RCSB protein data bank (https://www.rcsb.org/). The dataset for screening is publicly available from the ZINC website (https://zinc.docking.org/). The decoy dataset is obtained from Schrödinger and DUD-E database (http://dude.docking.org/). Active LDHA inhibitors were retrieved from the ChEMBL database (https://www.ebi.ac.uk/chembl/). Decoys were generated from the publicly available Decoyfinder software. 2D fingerprints were generated by the Canvas software purchased from Schrödinger, version 2020-3 (https://www.schrodinger.com/). Pharmacophore models and ROC curves were generated from the program Catalyst purchased from the Biovia Drug Discovery Studio, version 2021 (https://www.3ds.com/products-services/biovia/products/molecular-modeling-simulation/biovia-discovery-studio/). Docking was done using the glide docking package from Schrödinger 2020-3. MM-GBSA calculations were done using the Prime software available from Schrödinger 2020-3. The thermodynamics of waters were calculated using the WaterMap program from Schrödinger version 2022-2. Molecular dynamics simulations were carried out using the Desmond program available from Schrödinger 2022-2. Chemical structures were drawn using ChemDraw Professional version 15.1. The chemical structure transformation was done using MayaChemTools (http://www.mayachemtools.org/).

## Supporting Information Available

Computational virtual screening data, structures of the hits identified from virtual screening, and morphological changes in pancreatic cancer cells upon treatment with **ZINC13469319** (PDF)

## Abbreviations Used

LDHA: lactate dehydrogenase-A
OXPHOS: oxidative phosphorylation
PDAC: pancreatic ductal adenocarcinoma
MD: molecular dynamics
SIM: similarity
BEDROC: Boltzmann-enhanced discrimination of the receiver operating characteristic
AUC: area under the curve
ROC: receiver operation characteristics
XP: extra precision
MM-GBSA: molecular mechanics/generalized born surface area
DMSO: dimethyl sulfoxide
