# Supplementary material for: Exploration of a Large Virtual Chemical Space: Identification of Potent Inhibitors of Lactate Dehydrogenase-A against Pancreatic Cancer
Source: J Chem Inf Model. 2023 Jan 16;63(3):1028–43. doi: 10.1021/acs.jcim.2c01544 (PMC9930117; doi:10.1021/acs.jcim.2c01544)
Supplement: Supplementary file 1 — ci2c01544_si_001.pdf [file ci2c01544_si_001.pdf]

Exploration of a Large Virtual Chemical Space: Identification of Potent Inhibitors of Lactate Dehydrogenase-A Against Pancreatic Cancer

Horrick Sharma[1]*, Pragya Sharma[2], Uzziah Urquiza[2], Lerin R. Chastain[3], and Michael A. Ihnat[3]

[1]Department of Pharmaceutical Sciences, College of Pharmacy, Southwestern Oklahoma State University, Weatherford, Oklahoma 73096, USA.

[2]Department of Biological Sciences, Southwestern Oklahoma State University, Weatherford, Oklahoma, 73096, USA.

[3]Department of Pharmaceutical Sciences, College of Pharmacy, University of Oklahoma Health Sciences Center, Oklahoma City, Oklahoma 73117, USA.

*Correspondence: author, E-mail address: horrick.sharma@swosu.edu

Supplementary Information

Table S1. **LDHA inhibitors from literature used as actives in 2D fingerprint screening**

ChEMBL ID	LDHA IC_{50}/Ki (μM)	Structure
**2382401**	0.480	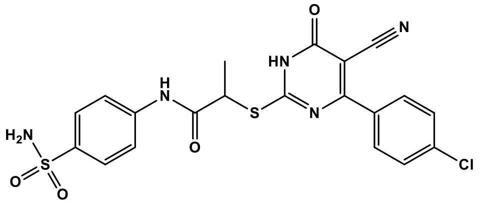
**2382404**	0.65	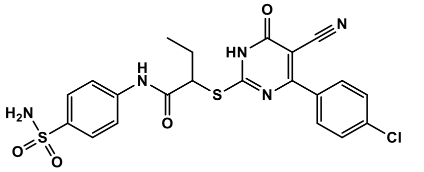
**2382403**	0.71	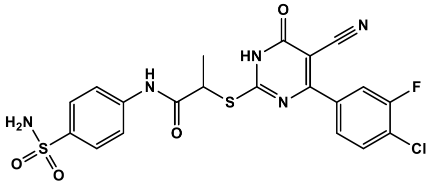
**2059811**	0.5	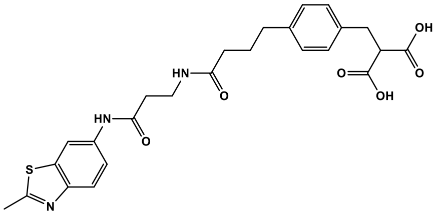

**3335792**	0.005	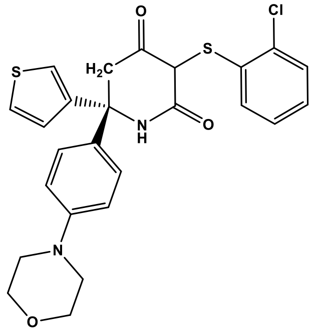
**3358864**	11.0	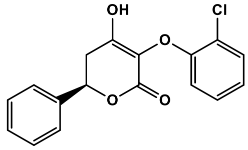
**3358866**	11.0	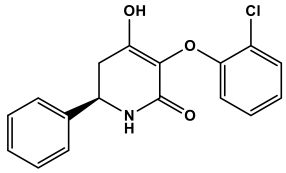
**3358863**	4.0	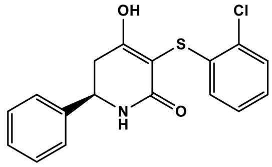
**3359439**	0.35	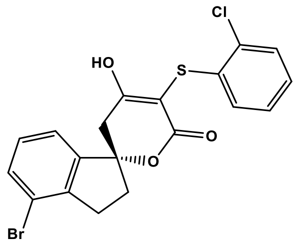
**3359440**	0.03	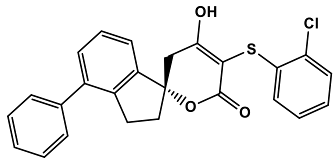
**3359438**	0.36	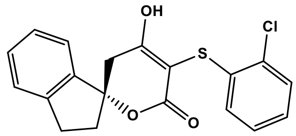

**3581201**	0.015	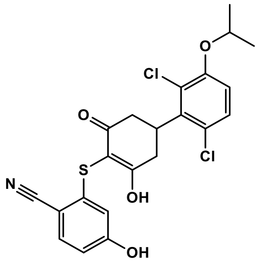
**3581199**	0.03	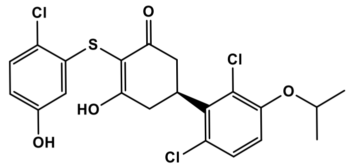
**3581200**	0.025	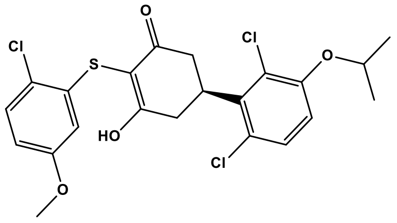
**3318538**	0.18	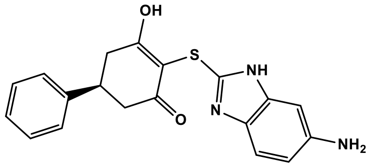
**3318527**	0.27	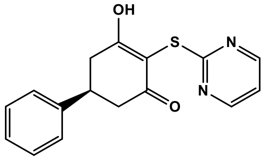
**3318535**	0.45	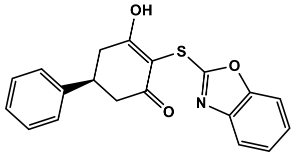
**2430719**	18.0	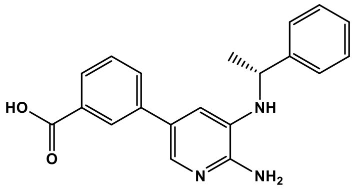
**2430727**	0.5	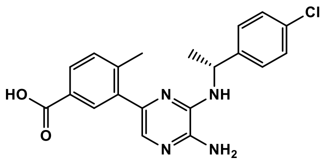

**2430733**	2.0	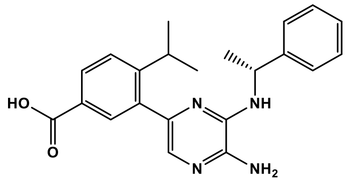
**2430734**	2.0	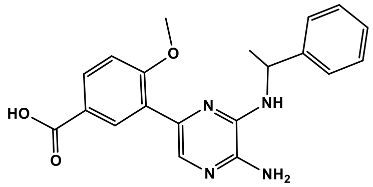
**1232973**	10.0	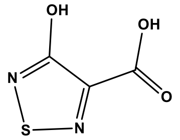
**3221028**	20.0	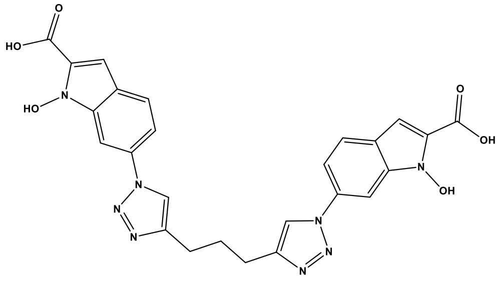
**1688788**	15.7	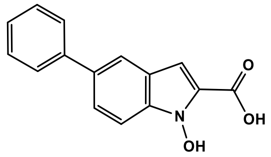
**1688789**	19.8	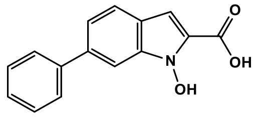
**1688790**	4.7	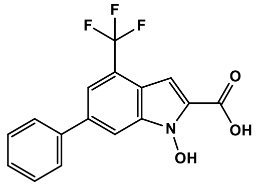
**3764862**	19.5	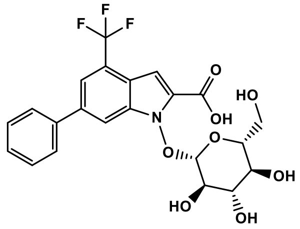

**3335796**	5.1	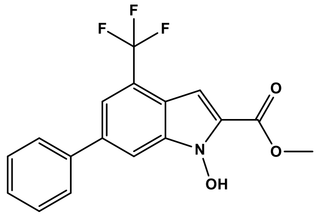

Table S2. Enrichment from method A using CHEMBL1232973 as the reference compound

S.No.	Active	Activity (IC_{50}/Ki) (μM)	Ranking of the Active	Ranking in Top 1%	Ranking in Top 5%	Ranking in Top 10%
1	**CHEMBL2382401**	0.48	65			*
2	**CHEMBL2382404**	0.65	66			*
3	**CHEMBL2382403**	0.71	67			*
4	**CHEMBL2059811**	0.50	68			*
5	**CHEMBL3335792**	0.005	69			*
6	**CHEMBL3358864**	1.10	70			*
7	**CHEMBL3358866**	1.10	71			*
8	**CHEMBL3358863**	4.00	72			*
9	**CHEMBL3359439**	0.35	73			*
10	**CHEMBL3359440**	0.03	74			*
11	**CHEMBL3359438**	0.36	75			*
12	**CHEMBL3581201**	0.015	76			*
13	**CHEMBL3581199**	0.030	77			*
14	**CHEMBL3581200**	0.025	78			*
15	**CHEMBL3318538**	0.18	79			*
16	**CHEMBL3318527**	0.27	80			*
17	**CHEMBL3318535**	0.45	81			*
18	**CHEMBL2430719**	18.0	18		*	*
19	**CHEMBL2430727**	0.50	34		*	*
20	**CHEMBL2430733**	2.0	30		*	*
21	**CHEMBL2430734**	2.0	35		*	*
22	**CHEMBL1232973**	10.0	1	*	*	*
23	**CHEMBL3221028**	20.0	8	*	*	*
24	**CHEMBL1688788**	15.7	5	*	*	*
25	**CHEMBL1688789**	19.8	4	*	*	*
26	**CHEMBL1688790**	4.7	6	*	*	*
27	**CHEMBL3764862**	19.5	12	*	*	*
28	**CHEMBL3335796**	5.10	82			*
Total no of actives retrieved in the Top 1, 5 and 10%				6	10	28

Table S3. Enrichment from data fusion using the 'MAX' rule from method B

S.No.	Active used as the reference	# Actives (Top 1%)	# Actives (Top 5%)	# Actives (Top 10%)
1	**CHEMBL2382401**	2	2	2
2	**CHEMBL2382404**	2	2	2
3	**CHEMBL2382403**	2	2	2
4	**CHEMBL2059811**	0	0	0
5	**CHEMBL3335792**	0	0	1
6	**CHEMBL3358864**	4	7	8
7	**CHEMBL3358866**	2	5	5
8	**CHEMBL3358863**	1	4	12
9	**CHEMBL3359439**	2	7	12
10	**CHEMBL3359440**	2	6	12
11	**CHEMBL3359438**	2	7	12
12	**CHEMBL3581201**	2	2	11
13	**CHEMBL3581199**	2	2	11
14	**CHEMBL3581200**	2	2	10
15	**CHEMBL3318538**	2	3	3
16	**CHEMBL3318527**	2	2	10
17	**CHEMBL3318535**	2	2	9
18	**CHEMBL2430719**	3	3	3
19	**CHEMBL2430727**	3	3	3
20	**CHEMBL2430733**	3	3	3
21	**CHEMBL2430734**	3	3	3
22	**CHEMBL1232973**	4	6	7
23	**CHEMBL3221028**	5	6	6
24	**CHEMBL1688788**	6	6	8
25	**CHEMBL1688789**	6	6	8
26	**CHEMBL1688790**	6	6	8
27	**CHEMBL3764862**	5	6	7
28	**CHEMBL3335796**	5	6	6

Table S4. Enrichment from data fusion using the 'SUM' rule from method B

S.No.	Active used as the reference	Number of Actives retrieved in the Top 1%	Number of Actives retrieved in the Top 5%	Number of Actives retrieved in the Top 10%
1	**CHEMBL2382401**	2	2	2
2	**CHEMBL2382404**	2	2	2
3	**CHEMBL2382403**	2	2	2
4	**CHEMBL2059811**	0	0	0
5	**CHEMBL3335792**	1	4	5
6	**CHEMBL3358864**	2	8	10
7	**CHEMBL3358866**	2	3	5
8	**CHEMBL3358863**	1	4	12
9	**CHEMBL3359439**	2	6	12
10	**CHEMBL3359440**	2	6	12
11	**CHEMBL3359438**	2	5	12
12	**CHEMBL3581201**	2	2	11
13	**CHEMBL3581199**	2	2	11
14	**CHEMBL3581200**	2	2	11
15	**CHEMBL3318538**	2	2	6
16	**CHEMBL3318527**	0	2	11
17	**CHEMBL3318535**	2	2	11
18	**CHEMBL2430719**	1	5	6
19	**CHEMBL2430727**	3	3	3
20	**CHEMBL2430733**	2	3	4
21	**CHEMBL2430734**	2	3	4
22	**CHEMBL1232973**	4	9	10
23	**CHEMBL3221028**	6	6	7
24	**CHEMBL1688788**	5	8	12
25	**CHEMBL1688789**	5	8	13
26	**CHEMBL1688790**	5	7	12
27	**CHEMBL3764862**	5	7	10
28	**CHEMBL3335796**	5	5	8

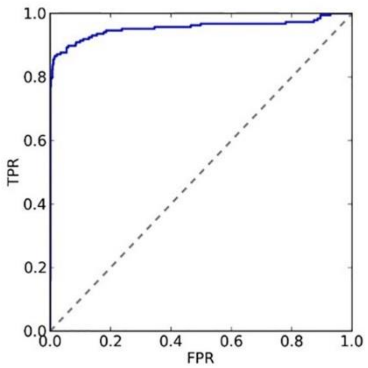

Figure S1. ROC plot from 2D fingerprint enrichment for the top 1% enrichment using method C.

Table S5. **Validation of the pharmacophore model B using 28 known LDHA inhibitors**

Pharmacophore	Total Actives	Total Inactives	True Positives	True Negatives	False Positives	False Negatives	Sensitivity	Specificity
Pharmacophore_1	82	1161	60	767	394	22	0.73171	0.66064
Pharmacophore_2	82	1161	74	741	420	8	0.90244	0.63824
Pharmacophore_3	82	1161	63	689	472	19	0.76829	0.59345
Pharmacophore_4	82	1161	70	680	481	12	0.85366	0.58570
Pharmacophore_5	82	1161	63	651	510	19	0.76829	0.56072
Pharmacophore_6	82	1161	63	625	536	19	0.76829	0.53833
Pharmacophore_7	82	1161	78	685	476	4	0.95122	0.59001
Pharmacophore_8	82	1161	66	606	555	16	0.80488	0.52196
Pharmacophore_9	82	1161	64	598	563	18	0.78049	0.51507
Pharmacophore_10	82	1161	78	581	580	4	0.95122	0.50043

Table S6. Validation of the receptor-based approach with method C built using receptor PDB ID 4ZVV

Pharmacophore	Total Actives	Total Inactives	True Positives	True Negatives	False Positives	False Negatives	Sensitivity	Specificity
Pharmacophore_1	123	1161	112	442	719	11	0.91057	0.38071
Pharmacophore_2	123	1161	110	400	761	13	0.89431	0.34453
Pharmacophore_3	123	1161	97	371	790	26	0.78862	0.31955
Pharmacophore_4	123	1161	113	427	734	10	0.91870	0.36779
Pharmacophore_5	123	1161	97	414	747	26	0.78862	0.35659
Pharmacophore_6	123	1161	97	367	794	26	0.78862	0.31611
Pharmacophore_7	123	1161	116	364	797	7	0.94309	0.31352
Pharmacophore_8	123	1161	101	184	977	22	0.82114	0.15848
Pharmacophore_9	123	1161	122	206	955	1	0.99187	0.17743
Pharmacophore_10	123	1161	121	171	990	2	0.98374	0.14729

Table S7. Validation of the receptor-based approach with method D built using receptor PDB ID 5IXY

Pharmacophore	Total actives	Total Inactives	True Positives	True Negatives	False Positives	False Negatives	Sensitivity	Specificity
Pharmacophore_1	104	1161	96	448	713	8	0.92308	0.38587
Pharmacophore_2	104	1161	96	396	765	8	0.92308	0.34109
Pharmacophore_3	104	1161	80	413	748	24	0.76923	0.35573
Pharmacophore_4	104	1161	93	425	736	11	0.89423	0.36606
Pharmacophore_5	104	1161	82	361	800	22	0.78846	0.31094
Pharmacophore_6	104	1161	82	364	797	22	0.78846	0.31352
Pharmacophore_7	104	1161	99	362	799	5	0.95192	0.31180
Pharmacophore_8	104	1161	86	190	971	18	0.82692	0.16365
Pharmacophore_9	104	1161	102	203	958	2	0.98077	0.17485
Pharmacophore_10	104	1161	103	177	984	1	0.99038	0.15245

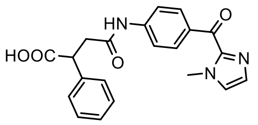

ZINC13469319

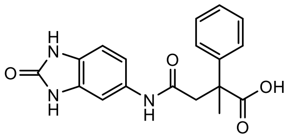

ZINC16481336

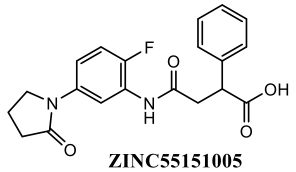

ZINC55151005

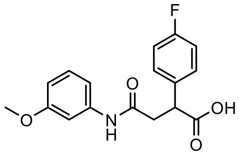

ZINC25204967

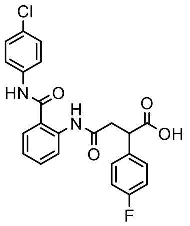

ZINC12817529

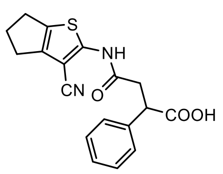

ZINC16482404

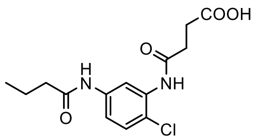

ZINC10287535

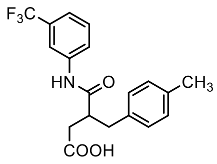

ZINC1162757

Figure S2. Structures of compounds with succinic acid monoamide moiety identified for biological testing.

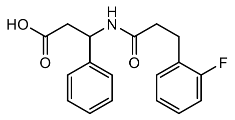

ZINC13225109

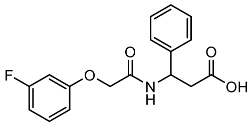

ZINC13224346

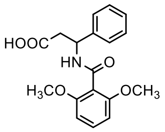

ZINC83975796

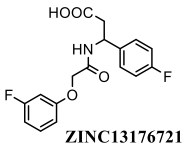

ZINC13176721

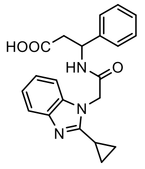

ZINC95424079

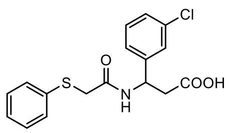

ZINC4686101

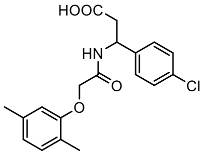

ZINC3626961

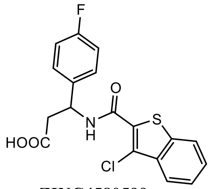

ZINC4580599

Figure S3. Structures of compounds with beta substitution and reverse amide moiety identified for biological testing.

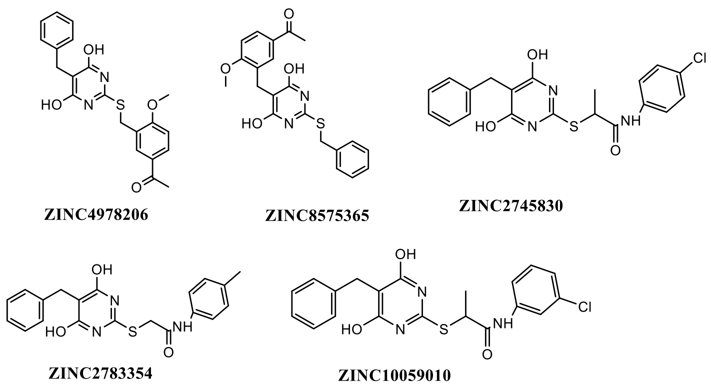

Figure S4. Structures of hydroxy pyrimidinones identified for biological testing.

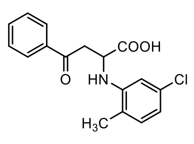

ZINC6278574

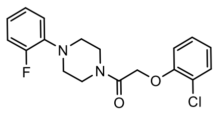

ZINC620615

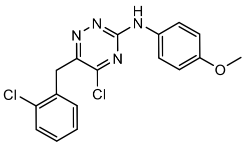

ZINC8579113

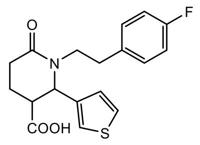

ZINC69492082

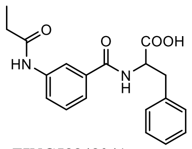

ZINC58848041

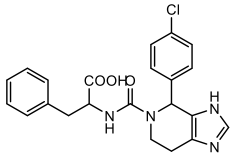

ZINC40267182

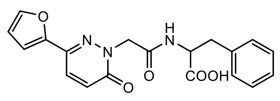

ZINC61718959

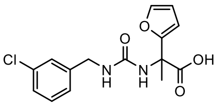

ZINC72260234

Figure S5. Miscellaneous compounds selected from virtual screening.

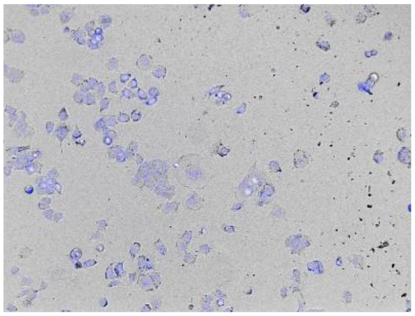

Panc, 0uM, D11, Field 1

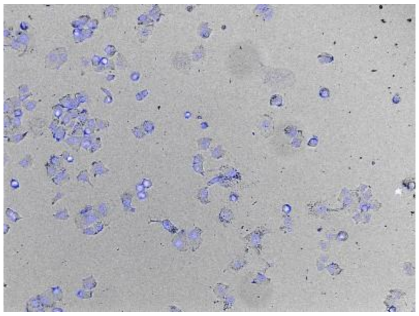

Panc, 0uM, B11, Field 1

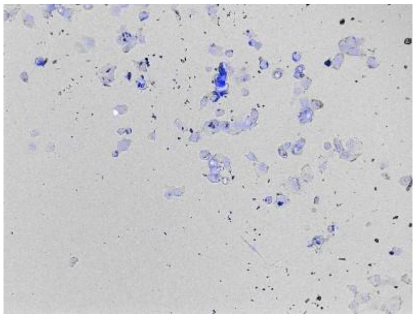

Panc, 25uM, D11, Field 1

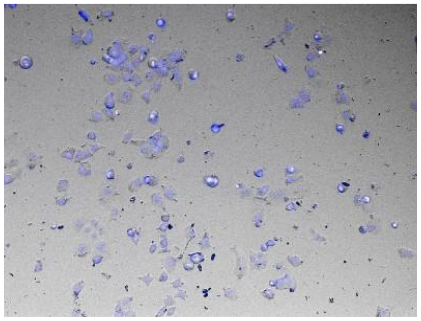

Panc, 25uM, DB2, Field 2

Figure S6. Morphological changes in PANC-1 cells upon treatment with ZINC13469319 vs control upon 48 hours.

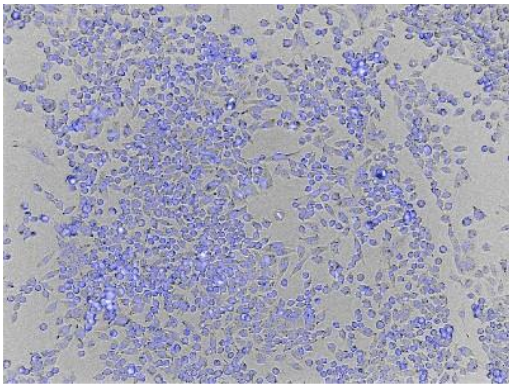

MiaPaca, 0uM, B11, Field 1

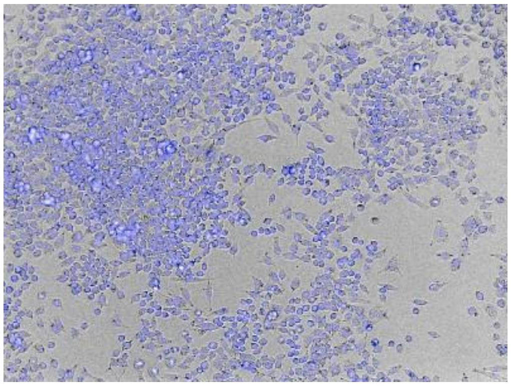

MiaPaca, 0uM, C11, Field 1

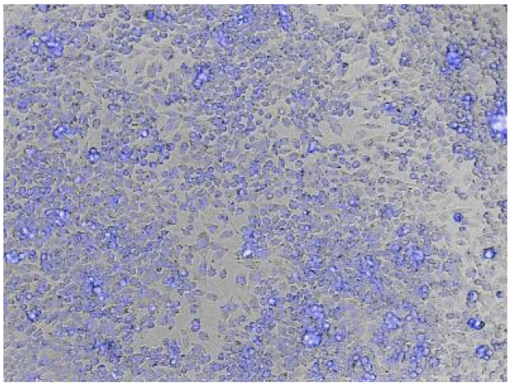

MiaPaca, 25uM, B2, Field 1

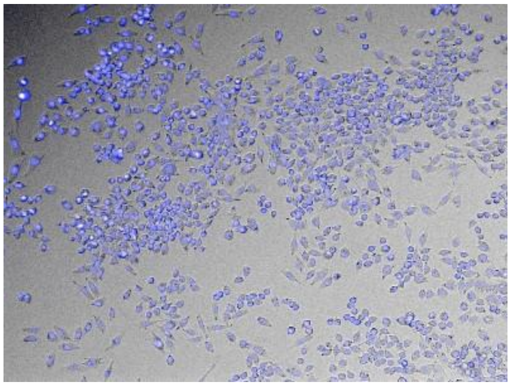

MiaPaca, 25uM, C2, Field 4

Figure S7. Morphological changes in MIA PaCa-2 cells upon treatment with ZINC13469319 vs control upon 48 hours.

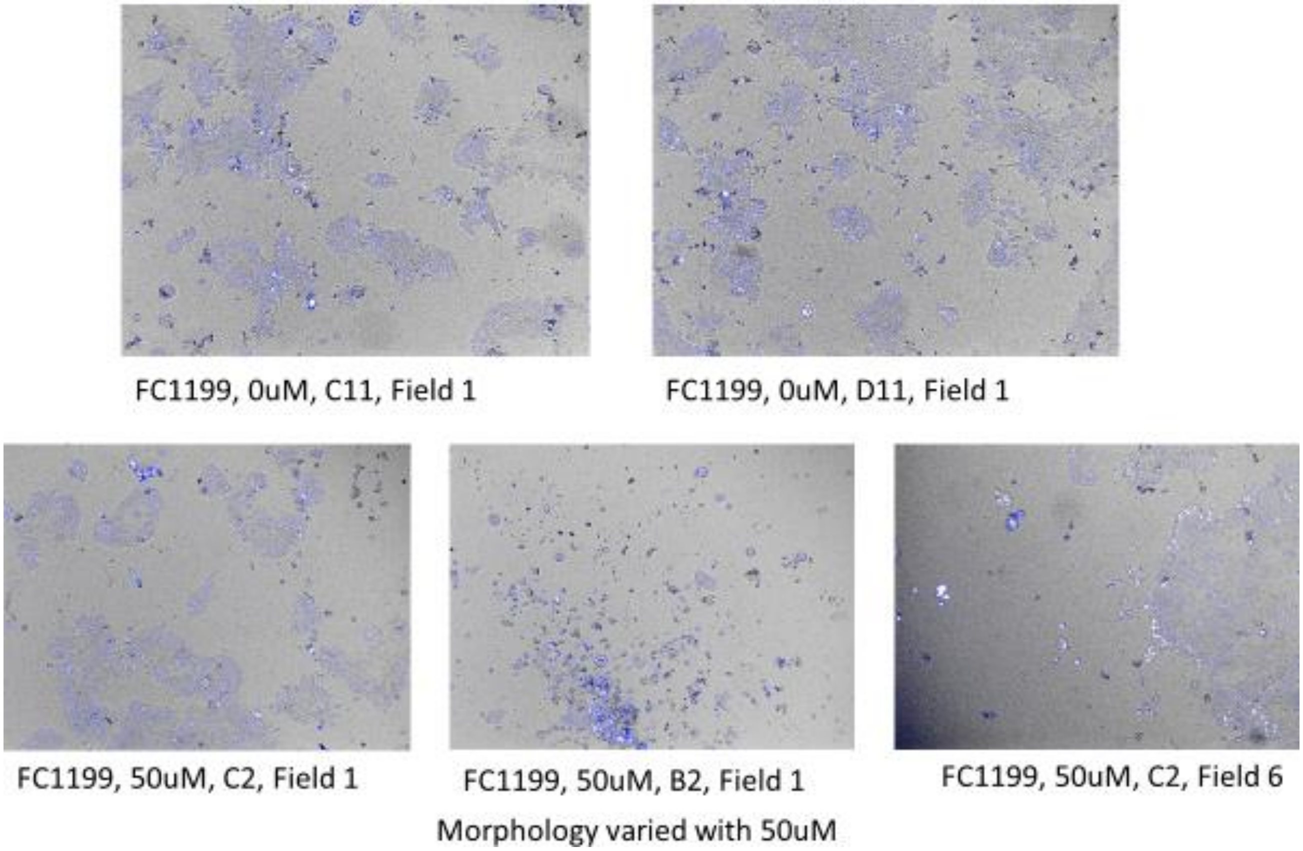

Figure S8. Morphological changes in FC1199 cells upon treatment with ZINC13469319 vs control upon 48 hours.
